# Separating the Role of Mixing‐Entropy on the Dynamics of Glass‐Forming Liquids

**DOI:** 10.1002/advs.202502568

**Published:** 2025-04-07

**Authors:** Qing‐Zhou Bu, Jia‐Xin Zhang, Hui‐Ru Zhang, Ran Li, Tao Zhang, Lin Liu, Peng Yu, Hai‐Bin Yu

**Affiliations:** ^1^ Wuhan National High Magnetic Field Center and School of Physics Huazhong University of Science and Technology Wuhan 430074 China; ^2^ Key Laboratory of Aerospace Materials and Performance (Ministry of Education) School of Materials Science and Engineering Beihang University Beijing 100191 China; ^3^ State Key Laboratory of Materials Processing and Die & Mould Technology School of Materials Science and Engineering Huazhong University of Science and Technology Wuhan 430074 China; ^4^ College of Physics and Electronic Engineering Chongqing Normal University Chongqing 401331 China

**Keywords:** fragility, high entropy alloy, metallic glass, mixing entropy

## Abstract

While the concept of high entropy has gained prominence in materials design, disentangling specific impacts of entropy on material properties from the enthalpy of mixing remains elusive. It is demonstrated that the role of entropy can be distinguished from the dynamics of glass‐forming liquids through micro‐alloying. Based on experiment analysis of 79 compositions, liquid fragility is found to consistently decreases under two conditions: i) when the alloying content *x* is minimal, irrespective of the elements used; or ii) when increasing the diversity of alloying elements at a constant *x*, namely the high‐entropy micro‐alloying. These observations are consistent with thermodynamic principles that favor an entropy‐dominated regime over enthalpy. These findings elucidate the subtle impact of mixing entropy on material properties and provide evidence of the entropy nature of glass transition.

## Graphical abstract

This research innovates by distinguishing entropy’s impact from enthalpy in materials through micro‐alloying. It finds liquid fragility decreases with minimal alloying content or increased element diversity. This clarifies entropy’s role and supports the entropy‐driven glass transition, guiding new material design.

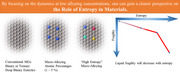

## 1 Introduction

High‐entropy materials, which incorporate at least five principal elements, represent a significant evolution from traditional materials based on one or two primary constituents.^[^ ^1^, ^2^, ^3^, ^4^, ^5^ ^]^ Since the advent of high‐entropy alloys (HEAs),^[^ ^6^, ^7^, ^8^ ^]^ entropy engineering has emerged as a cutting‐edge domain within materials science.^[^ ^1^, ^2^, ^3^, ^4^, ^5^, ^7^, ^9^, ^10^, ^11^, ^12^, ^13^, ^14^ ^]^ This paradigm leverages the concept of mixing entropy (Δ*S_mix_*) to design and fabricate materials.^[^ ^1^ ^]^

According to statistical thermodynamics, the mixing entropy is given $\Delta S_{mix}=-k_{B}\sum_{i}x_{i}\ln\left(x_{i}\right)$ where *x_i_* is the molar fraction of the *i*‐th component and subjected to $\sum_{i}x_{i}=1$. Typically, the high entropy is achieved by increasing the number of the component. Nonetheless, in practical materials, the mixing enthalpy also plays a role, expressed as Δ*H_mix_* = 4∑_(*A* ≠*B≠*)_ *H_AB_* ^*mix*^ *x_A_x_B_* where *A* and *B* are different elements. While Δ*S_mix_* quantifies the diversity of configurations due to mixing, Δ*H_mix_* reflects the interactions among the different constituent elements. Collectively, both factors influence the Gibbs free energy *G* = *H* −*TS*.

High‐entropy materials are promising materials that can provide outstanding properties, such as enhanced strength, excellent corrosion resistance, and improved thermal stability,^[^ ^15^, ^16^, ^17^, ^18^, ^19^, ^20^, ^21^, ^22^, ^23^, ^24^ ^]^ yet the precise roles of entropy and enthalpy in dictating material properties are still not fully disentangled. For example, Rost et al.^[^ ^25^ ^]^ reported a high‐entropy oxide Mg_0.2_Co_0.2_Ni_0.2_Cu_0.2_Zn_0.2_O and suggested that entropy predominantly facilitates homogeneous single‐phase formation. In contrast, Fracchia et al.^[^ ^26^ ^]^ demonstrated that configuration entropy is not the dominant factor in the thermodynamic stability of this oxide. Instead, the contents of CuO and ZnO are crucial, suggesting the contribution of mixing enthalpy.

Through a systematic analysis of the relative impacts of enthalpy and entropy on phase stability, Otto et al.^[^ ^12^ ^]^ concluded that enthalpy and non‐configurational entropy exert more significant influences on phase stability in equiatomic, multi‐component alloys. Additionally, chemical short‐range orders^[^ ^27^, ^28^ ^]^ have been observed in several high entropy alloys, which are attributed to the effects of mixing enthalpy.

In the case of amorphous high entropy alloys, Qiao et al.^[^ ^29^ ^]^ reported that a high entropy metallic glass Pd_20_Pt_20_Cu_20_Ni_20_P_20_ has a larger jump of heat capacity *ΔC_p_* at the glass transition *T_g_*; whereas, Yang et al.^[^ ^30^ ^]^ observed that *ΔC_p_* is much weaker in Zr_20_Ti_20_Cu_20_Ni_20_Be_20_ than a similar composition (Zr_41.2_Ti_13.8_Cu_12.5_Ni_10_Be_22.5_) with lower *ΔS_mix_*.

Moreover, Jiang et al.^[^ ^31^ ^]^ reported a decoupling between calorimetry and dynamic glass transition in high‐entropy metallic glasses. On the other hand, Li et al.^[^ ^32^ ^]^ used computational simulations to demonstrate that this decoupling is particularly prominent in alloys with significant differences in atomic size among elements, indicating the influence of both entropy and atomic size.

These seemingly contrasting examples collectively illustrate that the role of entropy in determining material properties is multifaceted and often intertwined with the complex interplay of various atomic‐scale interactions. Multiple elements with varying sizes and chemical affinities in high‐entropy materials can create a complicated a complex landscape of entropic and enthalpic contributions, making it challenging to isolate the pure effects of entropy.

The ambiguity surrounding the role of entropy in determining material properties has led to the introduction of alternative terms for high‐entropy alloy, such as “chemically complex alloy”, “concentrated alloy” and “multi‐principal element alloy”. This reflects a need to better understand and differentiate the contributions of entropy and enthalpy.

**Figure 1:**
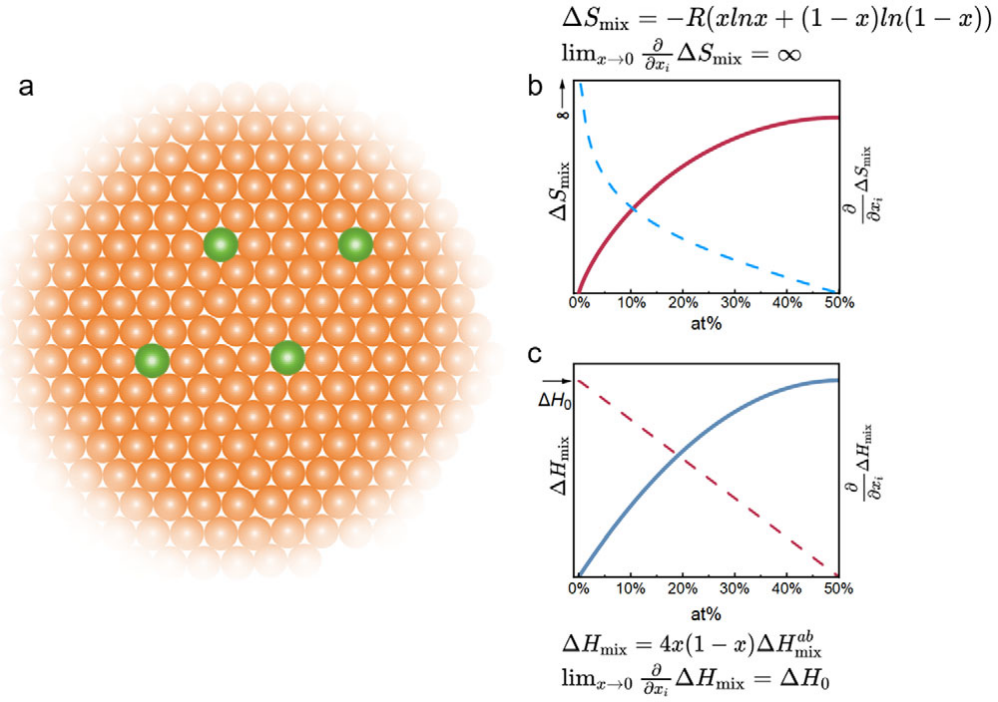
Mixing entropy and enthalpy. a) schematic for entropy effect b) mixing entropy and c) mixing enthalpy as a function of composition *x* and their derivatives.

In this study, we delve into the role of entropy by examining its influence on liquid dynamics by focusing on the effects of micro‐alloying. Our approach is grounded in the principles of thermodynamics. As illustrated in **Figure** **1**, the rate of change of the mixing entropy with respect to the concentration *x* approaches infinity as *x* tends toward zero, $\lim_{x\to 0}\frac{d\Delta S_{mix}}{dx}\;\to \infty$. In contrast, the rate of change of the mixing enthalpy remains finite $\lim_{x\to 0}\frac{d\Delta H_{mix}}{dx}=H_{0}\;$. Given this scenario, for sufficiently small values of *x*, it is reasonable to anticipate that any observed changes in material properties would predominantly be ascribed to the effects of entropy. By focusing on the dynamics at low alloying concentrations, one can gain a clearer perspective on the role of entropy in materials, which can inform the design of novel materials with tailored properties. We propose a “high entropy micro‐alloying” approach to investigate this scenario by maintaining a small value of x and increasing the diversity of alloying elements.

## 2 Methods

### 2.1 Sample Production

In this work, all the master alloy ingots were synthesized by melting high purity elements (purity ≥ 99.95 at%) under a Ti‐gettered argon atmosphere in an arc‐melting furnace. The ingots were flipped and remelted five times to ensure compositional homogeneity. Amorphous ribbons were produced by melt‐spinning method with a single‐copper roller at a wheel surface velocity of 50–65 m s^−1^ under a high‐purity argon atmosphere. The glassy nature of all the ribbons was verified using a Bruker D2 Phaser X‐ray diffractometer (XRD) with Cu Kα radiation (λ = 1.542 Å) at room temperature, employing a scanning rate of 5 ° min^−1^ under θ – 2θ mode. The Thermo Fisher Scios 2 dual‐beam focused ion beam scanning electron microscope (FIB‐SEM) equipped with an Energy‐Dispersive X‐ray Spectrometer (EDS) was employed to analyze the microstructural morphology and elemental distribution of the sample. The relevant data can be found in the Supporting information.

### 2.2 Calorimetry Measurement and Fragility Determining

Heat flow curves for the samples were obtained using a combination of Flash Differential Scanning Calorimetry (FSC) and Differential Scanning Calorimetry (DSC). For Cu‐Zr‐based metallic glasses (MGs), conventional DSC was employed to measure the heat flow curves, considering their excellent glass forming ability and pronounced supercooled liquid region. The conventional DSC heat flow curves were measured with a Mettler Toledo DSC 3 under a nitrogen atmosphere with a flow rate of 50 mL min^−1^. FSC (Mettler Toledo Flash DSC 2+) measurement was conducted for samples that exhibit relatively narrow supercooled liquid regions with an argon gas flows of 80 mL min^−1^. Each sample's heat flow curve was measured three times to ensure data reliability. The FSC chip sensors were preconditioned and calibrated following the manufacturer's recommendations.

**Figure 2:**
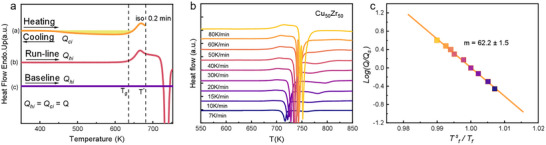
Curves for fragility determining by the Wang‐Velikov‐Angell (WVA) method. a) Schematic diagram of the scanning steps of DSC. b) Heat flow curves of a Cu_50_Zr_50_ MG with different heating rates. c) Fictive temperature (*T_f_*) dependence of logarithm heating rate Q. The slope of the fitted solid line reflects the magnitude of fragility.

**Figure** **2**a shows heat flow curves of a Cu_50_Zr_50_ MG for fragility determining. The sample was heated in the DSC to 680 K (T*) and held for 12 s. Then cooled down to 300 K at a selected cooling rate. An up‐scan through *T_g_* at the same rate was performed immediately (run‐line). The crystallized sample was cooled and re‐scanned up again at the same rate (baseline).^[^ ^33^ ^]^ The same procedure was performed with different cooling and heating rates Q from 7 to 80 K min^−1^ as shown in Figure 2b. Fragility determining was based on the heating rate dependence of T*_g_*, as shown in Figure 2c by the Wang‐Velikov‐Angell (WVA) method.^[^ ^34^ ^]^ The fragility of all samples was obtained through the previously mentioned measurement and fitting methods. In the supplementary information, the details on the experimental setup and fitted curves for the metallic glasses mentioned in this work are provided.

## 3 Results

Metallic glasses represent ideal models for studying homogeneous mixtures, avoiding the complexities of lattice structures, defects and phase separations found in crystalline materials. We focus on the dynamics of the glass transition, particularly liquid fragility, defined as

(1) $$m=d\log\left(\varphi \right)/d\left(T_{g,ref}/T\right){|}_{T=T_{g,ref}}$$

where φ is a dynamic parameter such as viscosity, relaxation time, or heating rate for a specified *T_g_*. It is a key concept in the science of glasses and liquids, reflecting the deviation of viscosity from Arrhenius behavior and impacting various glass properties. While theories suggest a link between glass transition and configuration entropy,^[^ ^35^, ^36^, ^37^ ^]^ the precise relationship is yet to be fully established by experiment. We measure fragility by a calorimetry approach based on the protocol of Evenson et al,^[^ ^38^ ^]^ which can yield fragility values that are consistent with viscosity measurements. We have recently employed this technique to uncover the subtle fragility crossover effects attributed to electronic interactions.^[^ ^39^ ^]^

**Figure 3:**
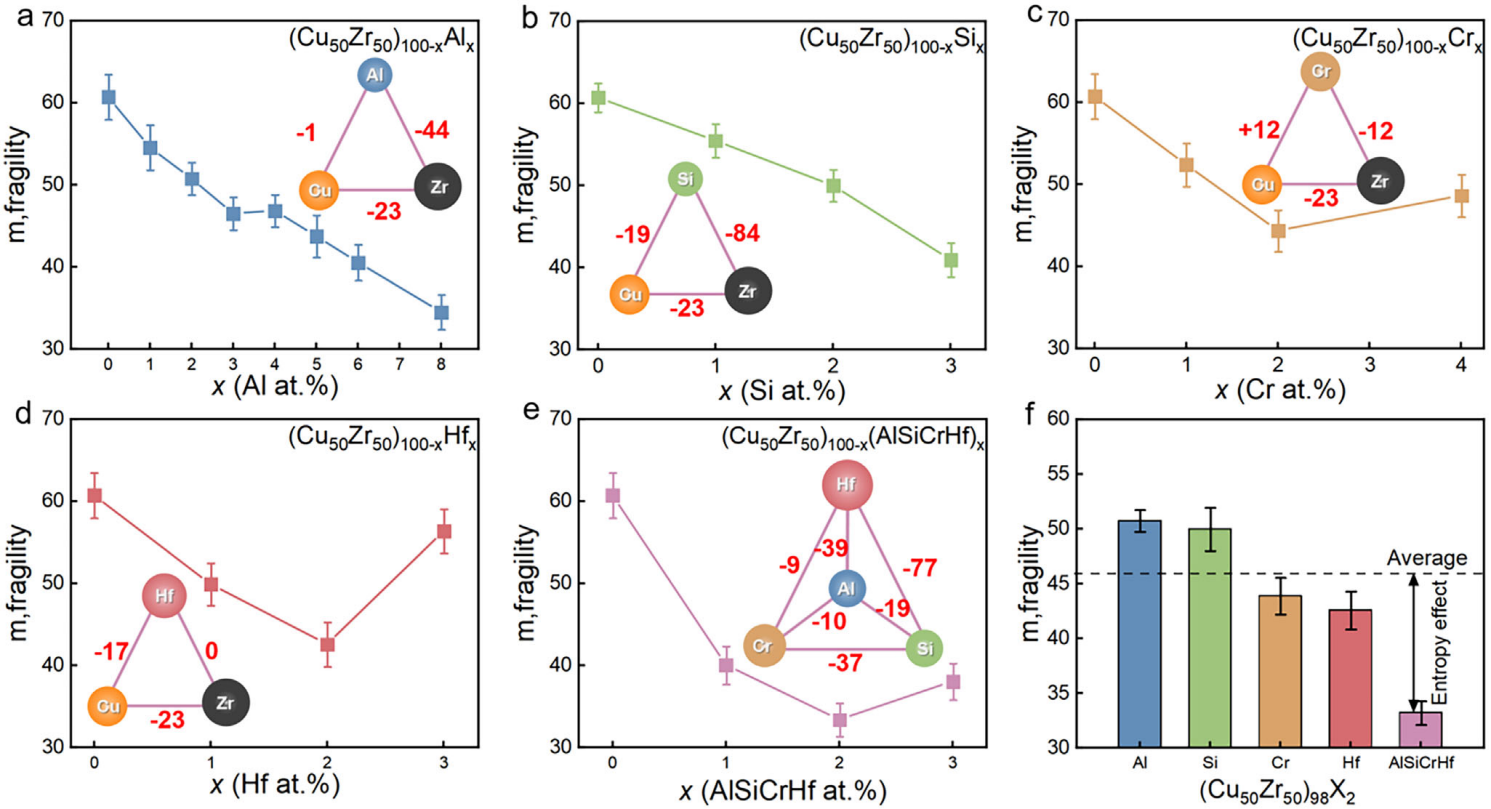
Alloying effects on fragility. a) (Cu_50_Zr_50_)_100‐_ *_x_* Al*_x_* b) (Cu_50_Zr_50_)_100‐_ *_x_* Si*_x_* c) (Cu_50_Zr_50_)_100‐_ *_x_* Cr*_x_* d) (Cu_50_Zr_50_)_100‐_ *_x_* Hf*_x_* e) (Cu_50_Zr_50_)_100‐_ *_x_* (AlSiCrHf)*_x_* f) fragility of (Cu_50_Zr_50_)_98_X_2_ for X = Al, Si, Cr, Hf and the AlSiCrHf with each elements having the equal molar fraction.

**Figure** **3** shows the typical results for alloying *X* in a Cu_50_Zr_50_ metallic glass with different molar fractions *x* in a composition formula (Cu_50_Zr_50_)_100‐_ *_x_* X*_x_*. For X = Al, it is observed that the fragility *m* decreases monotonically with *x* ≤ 8, aligning with earlier findings.^[^ ^40^ ^]^ A similar pattern is noted for X = Si up to x ≤ 3. On the other hand, for X = Cr or Hf, *m* initially decreases with x but rises when *x* > 23. Admittedly, the change in *m* depends on both the chemical properties of X and its molar fraction *x*.

It is interesting to summarize a unified pattern that when the alloying molar fraction is small *x* →0, *m* always decreases with *x*, irrespective of the chemical nature of *X*. We have confirmed this finding with other alloying elements such as X = Ni, Ag, and Ge in the Cu‐Zr system.^[^ ^41^ ^]^ These additional data can be found in **Table** **1**.

**Table 1:** The Tg, m, ΔHmix, S_C_, and S_E_ of studied MGs.

ID	Composition	T_g_ [K]	m	△H_mix_ [kJ mol^−1^]	*S_C_* [J/mol K]	S*_E_* [J/mol K]
**1**.	Cu_50_Zr_50_	680.0	60.7	−23.00	5.76	−3.44
**2**.	(Cu_50_Zr_50_)_99_Ag_1_	678.7	53.8	−22.90	6.17	−3.41
**3**.	(Cu_50_Zr_50_)_98_Ag_2_	683.6	49.6	−22.79	6.46	−3.37
**4**.	(Cu_50_Zr_50_)_97_Ag_3_	680.9	38.4	−22.69	6.71	−3.34
**5**.	(Cu_50_Zr_50_)_96_Ag_4_	685.0	35.6	−22.58	6.93	−3.31
**6**.	(Cu_50_Zr_50_)_95_Ag_5_	682.1	39.1	−22.47	7.13	−3.27
**7**.	(Cu_50_Zr_50_)_94_Ag_6_	682.5	44.3	−22.35	7.30	−3.24
**8**.	(Cu_50_Zr_50_)_93_Ag_7_	681	44.5	−22.24	7.47	−3.21
**9**.	(Cu_50_Zr_50_)_92_Ag_8_	681.4	52.3	−22.12	7.62	−3.18
**10**.	(Cu_50_Zr_50_)_99_Al_1_	681.1	54.6	−23.43	6.17	−3.41
**11**.	(Cu_50_Zr_50_)_98_Al_2_	694.3	50.7	−23.85	6.46	−3.38
**12**.	(Cu_50_Zr_50_)_97_Al_3_	699.1	47.4	−24.26	6.71	−3.34
**13**.	(Cu_50_Zr_50_)_96_Al_4_	698.4	46.8	−24.65	6.93	−3.31
**14**.	(Cu_50_Zr_50_)_95_Al_5_	701.6	43.7	−25.03	7.13	−3.28
**15**.	(Cu_50_Zr_50_)_94_Al_6_	704.0	40.5	−25.40	7.30	−3.25
**16**.	(Cu_50_Zr_50_)_92_Al_8_	708.1	34.5	−26.09	7.62	−3.18
**17**.	(Cu_50_Zr_50_)_99_Si_1_	705.7	55.5	−24.58	6.17	−3.56
**18**.	(Cu_50_Zr_50_)_98_Si_2_	711.1	49.9	−26.13	6.46	−3.67
**19**.	(Cu_50_Zr_50_)_97_Si_3_	709.5	40.9	−27.64	6.71	−3.78
**20**.	(Cu_50_Zr_50_)_99_Cr_1_	691.4	52.4	−22.54	6.17	−3.45
**21**.	(Cu_50_Zr_50_)_98_Cr_2_	684.2	44.3	−22.09	6.46	−3.46
**22**.	(Cu_50_Zr_50_)_96_Cr_4_	680.2	48.6	−21.20	6.93	−3.48
**23**.	(Cu_50_Zr_50_)_99_Hf_1_	693.6	49.9	−22.88	6.17	−3.42
**24**.	(Cu_50_Zr_50_)_98_Hf_2_	689.9	42.6	−22.76	6.46	−3.41
**25**.	(Cu_50_Zr_50_)_97_Hf_3_	680.6	56.4	−22.63	6.71	−3.39
**26**.	(Cu_50_Zr_50_)_98_Ni_2_	687.4	44.8	−23.85	6.46	−3.50
**27**.	(Cu_50_Zr_50_)_98_(AlSi)_2_	697.5	49.7	−25.00	6.58	−3.52
**28**.	(Cu_50_Zr_50_)_98_(HfSi)_2_	691.3	46.1	−24.47	6.58	−3.54
**29**.	(Cu_50_Zr_50_)_98_(AlHf)_2_	698.0	41.6	−23.32	6.58	−3.39
**30**.	(Cu_50_Zr_50_)_98_(CrHf)_2_	685.8	40.2	−22.43	6.58	−3.44
**31**.	(Cu_50_Zr_50_)_98_(AlCr)_2_	700.2	42.3	−22.98	6.58	−3.42
**32**.	(Cu_50_Zr_50_)_98_(NiCr)_2_	682.3	41.2	−22.97	6.58	−3.48
**33**.	(Cu_50_Zr_50_)_98_(NiHf)_2_	687.0	40.6	−23.32	6.58	−3.46
**34**.	(Cu_50_Zr_50_)_98_(NiAl)_2_	687.6	42.2	−23.86	6.58	−3.44
**35**.	(Cu_50_Zr_50_)_98_(HfAlSi)_2_	692.5	46.6	−24.34	6.64	−3.49
**36**.	(Cu_50_Zr_50_)_98_(CrAlSi)_2_	710.4	44.9	−24.13	6.64	−3.50
**37**.	(Cu_50_Zr_50_)_98_(CrHfSi)_2_	700.5	37.6	−23.76	6.64	−3.52
**38**.	(Cu_50_Zr_50_)_98_(CrHfAl)_2_	675.7	37.2	−22.95	6.64	−3.41
**39**.	(Cu_50_Zr_50_)_98_(NiCrHf)_2_	679.7	37.4	−22.86	6.64	−3.46
**40**.	(Cu_50_Zr_50_)_98_(NiCrAl)_2_	679.2	38.9	−23.24	6.64	−3.44
**41**.	(Cu_50_Zr_50_)_98_(NiCrSi)_2_	676.1	40.1	−24.05	6.64	−3.55
**42**.	(Cu_50_Zr_50_)_98_(NiHfAl)_2_	672.9	36.8	−23.49	6.64	−3.43
**43**.	(Cu_50_Zr_50_)_98_(NiHfSi)_2_	685.9	42.8	−24.29	6.64	−3.53
**44**.	(Cu_50_Zr_50_)_98_(NiAlSi)_2_	681.7	42.5	−24.66	6.64	−3.52
**45**.	(Cu_50_Zr_50_)_99_(AlSiCrHf)_1_	694.8	40.0	−23.36	6.29	−3.46
**46**.	(Cu_50_Zr_50_)_98_(AlSiCrHf)_2_	695.5	33.4	−23.73	6.69	−3.48
**47**.	(Cu_50_Zr_50_)_99_(NiCrHfAl)_2_	675.3	36.1	−23.15	6.69	−3.44
**48**.	(Cu_50_Zr_50_)_99_(NiCrHfSi)_2_	713.7	36.8	−23.73	6.69	−3.51
**49**.	(Cu_50_Zr_50_)_99_(NiCrAlSi)_2_	686.7	37.3	−23.99	6.69	−3.50
**50**.	(Cu_50_Zr_50_)_99_(NiHfAlSi)_2_	697.7	37.3	−24.17	6.69	−3.49
**51**.	(Cu_50_Zr_50_)_97_(CrHfAlSi)_3_	692.4	38.1	−24.08	7.06	−3.50
**52**.	(Cu_50_Zr_50_)_98_(CrHfAlSiNi)_2_	700.4	34.3	−23.75	6.73	−3.48
**53**.	Y_65_Co_35_	600.7	53.0	−20.02	5.38	N/A
**54**.	(Y_65_Co_35_)_99_Zr_1_	600.9	49.4	−19.96	5.79	N/A
**55**.	(Y_65_Co_35_)_98_Zr_2_	602.9	44.2	−19.89	6.09	N/A
**56**.	(Y_65_Co_35_)_97_Zr_3_	605.4	41.3	−19.83	6.34	N/A
**57**.	(Y_65_Co_35_)_99_La_1_	594.6	48.9	−19.86	5.79	N/A
**58**.	(Y_65_Co_35_)_98_La_2_	591.2	45.8	−19.69	6.09	N/A
**59**.	(Y_65_Co_35_)_97_La_3_	587.2	48.6	−19.53	6.34	N/A
**60**.	(Y_65_Co_35_)_99_Al_1_	600.1	49.0	−20.86	5.79	N/A
**61**.	(Y_65_Co_35_)_98_Al_2_	600.0	43.7	−21.69	6.09	N/A
**62**.	(Y_65_Co_35_)_97_Al_3_	600.0	40.4	−22.49	6.34	N/A
**63**.	(Y_65_Co_35_)_99.5_Te_0.5_	605.8	52.2	N/A	5.39	N/A
**64**.	(Y_65_Co_35_)_99_Te_1_	611.9	52.0	N/A	5.79	N/A
**65**.	(Y_65_Co_35_)_98_Te_2_	621.7	45.6	N/A	6.09	N/A
**66**.	(Y_65_Co_35_)_97_Te_3_	629.6	59.9	N/A	6.34	N/A
**67**.	(Y_65_Co_35_)_99_(AlZrTeLa)_1_	598.5	45.4	N/A	5.91	N/A
**68**.	(Y_65_Co_35_)_98_(AlZrTeLa)_2_	606.7	39.9	N/A	6.17	N/A
**69**.	(Y_65_Co_35_)_97_(AlZrTeLa)_3_	642.3	46.2	N/A	6.68	N/A
**70**.	La_50_Ni_25_Al_25_	486.2	35.5	−38	8.64	−7.64
**71**.	La_50_Cu_25_Al_25_	457.6	34.7	−29.75	8.64	−6.97
**72**.	Ce_50_Ni_25_Al_25_	492.2	33.4	−38.5	8.64	−6.64
**73**.	Ce_50_Cu_25_Al_25_	459.5	32.4	−29.75	8.64	−5.99
**74**.	La_25_Ce_25_Ni_12.5_Cu_12.5_Al_25_	465.6	28.6	−33.75	12.96	−6.84
**75**.	Gd_55_Ni_20_Al_25_	585.1	36	−39.49	8.29	−5.63
**76**.	Gd_55_Co_20_Al_25_	594.4	37.1	−34.93	8.29	−5.49
**77**.	Dy_55_Ni_20_Al_25_	622.5	32.9	−39.38	8.29	−5.28
**78**.	Dy_55_Co_20_Al_25_	627.9	32.1	−34.82	8.29	−5.15
**79**.	Gd_27.5_Dy_27.5_Ni_10_Co_10_Al_25_	609.1	29.1	−37.16	12.61	−5.39

N/A: There is no reliable experimental data on mixing enthalpy and atomic radius.

Additionally, we note that *m* (x) also depends on the mixing enthalpy, as shown in Figure 3c,d, *m* increases when *x* is not small, which might be due to positive mixing enthalpy.

**Figure 4:**
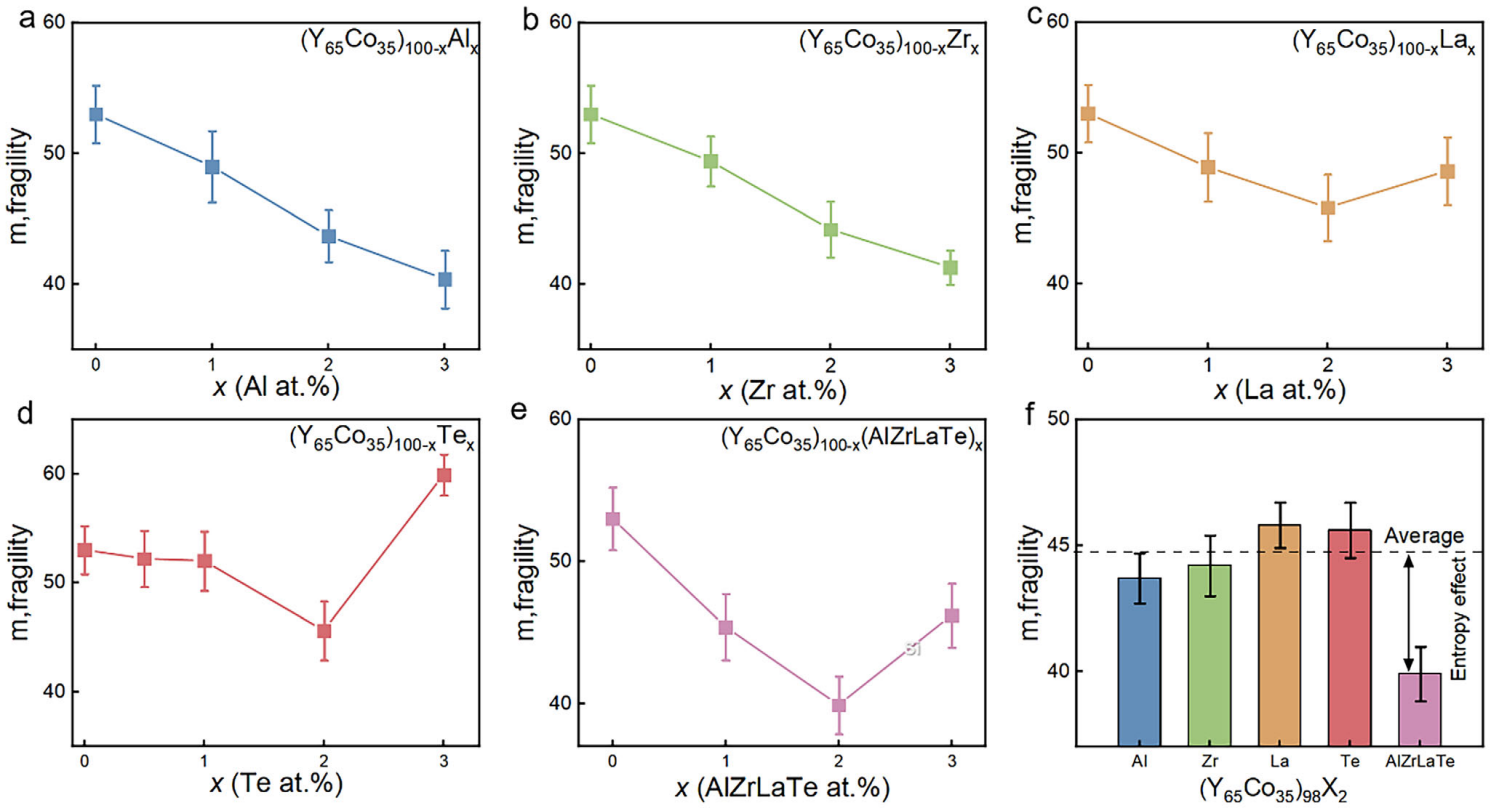
Alloying effects on fragility. a) (Y_65_Co_35_)_100‐_ *_x_* Al*_x_* b) (Y_65_Co_35_)_100‐_ *_x_* Zr*_x_* c) (Y_65_Co_35_)_100‐_ *_x_* La*_x_* d) (Y_65_Co_35_)_100‐_ *_x_* Te*_x_* e) (Y_65_Co_35_)_100‐_ *_x_* (AlZrLaTe)*_x_* f) fragility of (Y_65_Co_35_)_98_X_2_ for X = Al, Zr, La, Te and the AlZrLaTe with each elements having the equal molar fraction.

To validate this finding, similar experiments were conducted in the Y‐Co system. Considering its lower glass‐forming ability compared to Cu‐Zr system, which makes the composition more likely to crystallize during micro‐alloying and hinders the accurate assessment of fragility,^[^ ^42^, ^43^ ^]^ the concentration x was limited to within 3, and heat flow curves were obtained using a Flash DSC. The details can be found in methods. For (Y_65_Co_35_)_100‐_ *_x_* X*_x_* where X = Al, Zr, Te, and La, **Figure** **4** shows a trend similar to that observed in Cu‐Zr system. As discussed in the introduction, as *x* approaches 0, entropy has a greater influence than enthalpy. Thus, these results indicate that liquid fragility will decrease with entropy. It is worth noting that we made an effort to select elements with entirely different characteristics, such as elemental periods, atomic sizes, electronic structures. This approach is intended to demonstrate that the observed effect is universal and depends solely on the molar fraction of the added elements.

Figures 3e and 4e illustrate the value of *m* for the “high entropy micro‐alloying” scenario, where X represents a mixture of (AlSiCrHf) and (AlZrLaTe) with equal molar fractions for each element, collectively accounting for *x*% of the total composition. We observe that *m* values for these compositions are significantly lower than those estimated by averaging the *m* values of the individual element alloyed composition. For instance, as depicted in Figure 3f, when *x* = 2, the high entropy sample (Cu_50_Zr_50_)_98_(AlSiCrHf)_2_ exhibits *m* = 34. In contrast, averaging the *m* values of the four individual compositions [(Cu_50_Zr_50_)_98_X_2_, with X being Al, Si, Cr, or Hf] gives a predicted *m_predict_* = 45. Consequently, the discrepancy between the measured *m* and the predicted *m_predict_* is attributed to the entropy effect.

We define *δm* = *m* − *m_predict_* as the entropy‐suppressed fragility resulting from the micro‐alloying of two or more elements, using the *m* value of a single‐element‐doped alloy to calculate *m_predict_*. For instance, in the case of (Cu_50_Zr_50_)_98_Al_2_ with *m* = 50.7 and (Cu_50_Zr_50_)_98_Cr_2_ with *m* = 44.3, one predicts the fragility of (Cu_50_Zr_50_)_98_(AlCr)_2_ as *m_predict_* = (50.7 + 44.32)/2 = 47.5 based on their average value. The experiment measured fragility *m* = 42.3 yields an entropy‐suppressed fragility *δm* = 42.3 − 47.5 = −5.2 for the (Cu_50_Zr_50_)_98_(AlCr)_2._

Meanwhile, we use δΔS = ΔS(*x*) – ΔS(*x* = 0) as the increased entropy due to micro‐alloying. Here, *ΔS* includes both the ideal mixing entropy and the entropy adjustments for atomic size effects, as recent studies^[^ ^44^, ^45^ ^]^ have indicated that the atomic size correction is also crucial.

**Figure 5:**
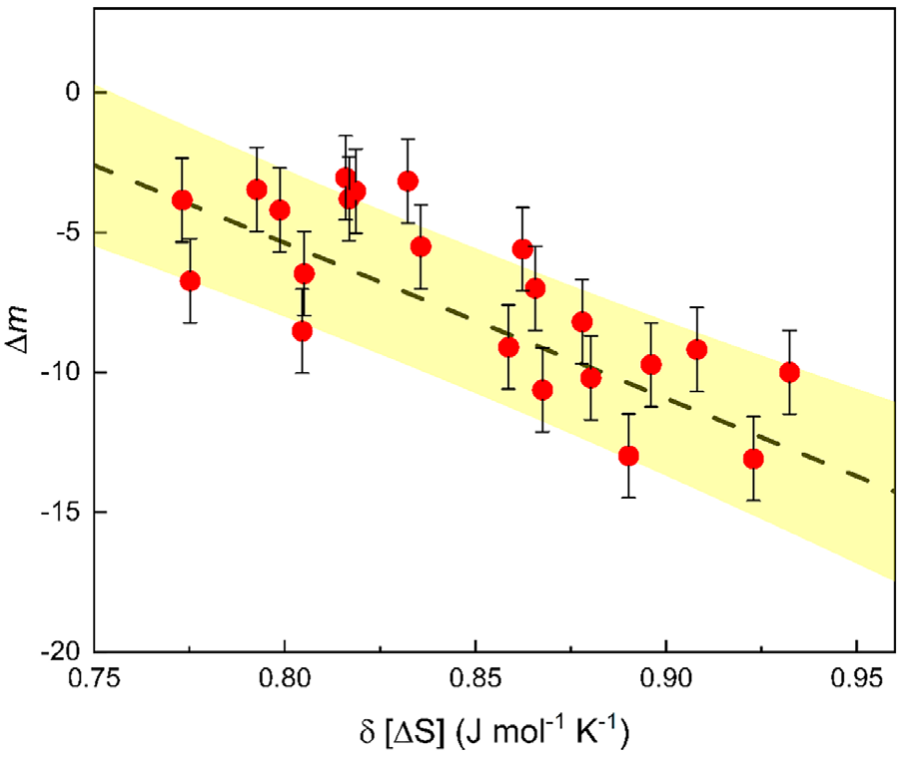
Relation between fragility change Δ*m* and alloying induced entropy change δΔS due to multiple elements alloying for the Cu_50_Zr_50_‐based metallic glasses.

**Figure** **5** plots δ*m* as a function δΔS for the (Cu_50_Zr_50_)_100‐_ *_x_* X*x* system with X stands for at least two elements in the collection of Al, Si, Cr, Hf, and Ni. We find that all *δm* < 0, which validates the *existence* of the entropy effect on liquid fragility. Moreover, the data reveals a decreasing trend for the two variables, suggesting that higher entropy leads to a more significant reduction in fragility. The results for all the studied Cu‐Zr‐based MGs with different compositions are collectively presented in Table 1, where their chemical composition, the glass transition temperature *T_g_*, the fragility m, the mixing enthalpy *ΔH_mix_*, the configuration entropy of mixing S*_C_*, and excess configuration entropy related to the packing density S*_E_* are presented in each row. These results provide clear‐cut evidence that reinforces the role of entropy in liquid dynamics.

**Figure 6:**
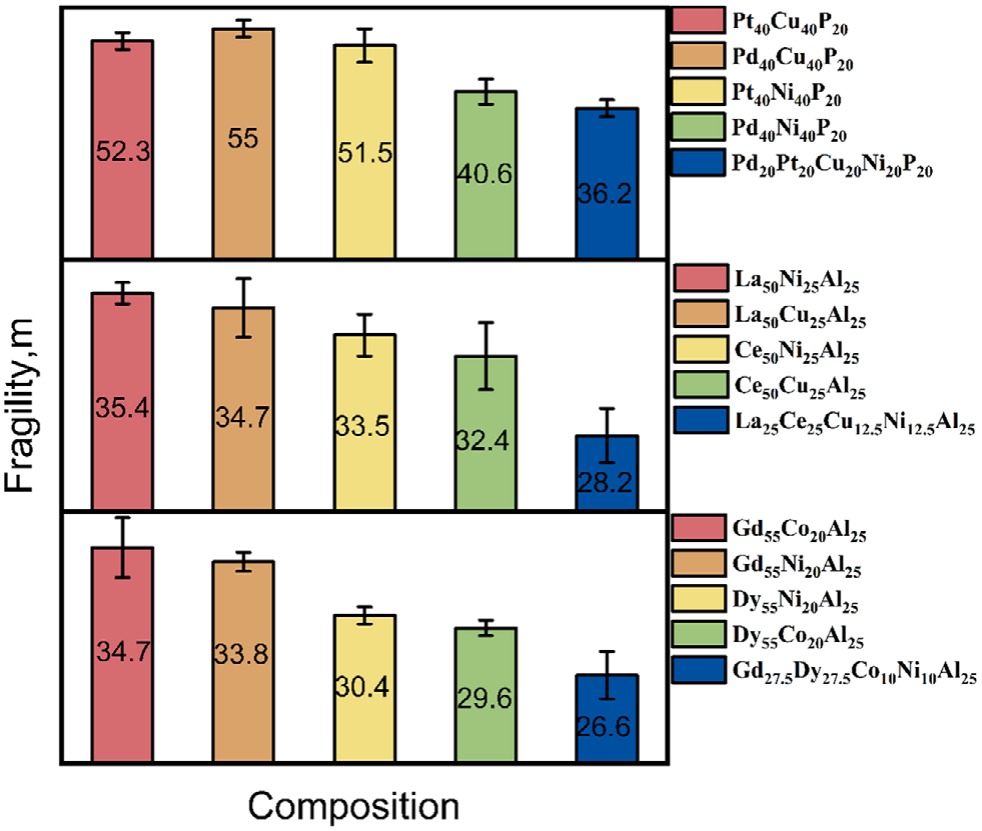
Fragility variation between high entropy metallic glasses and their related ternary metallic glasses. The numbers in the figure represent the fragility values obtained from Arrhenius fitting, and the legend on the right specifies the corresponding compositions.

We extend our study from micro‐alloying to conventional high‐entropy materials that contain multiple elements with high concentrations. It is worth noting that during this extension process, it is essential to ensure that factors such as mixing enthalpy and atomic radius do not cause significant interference. The high‐entropy metallic glass (HEMGs) systems we selected were derived from existing metallic glass (MGs) systems by substituting elements from the same group or with similar chemical properties. This approach ensured that neither the mixing enthalpy nor the atomic radii underwent significant changes. For instance, the $\Delta H_{AB}^{mix}$ for La‐Ce, Dy‐Gd, atomic pairs are 0 kJ mol^−1^, while the atomic radii exhibited only negligible differences as well.^[^ ^46^ ^]^ **Figure** **6** presents our experiment results. As a typical example, Figure 6 shows that the high entropy metallic glasses La_25_Ce_25_Ni_12.5_Cu_12.5_Al_25_ has *m* = 28.6. This value is lower than any of its related ternary metallic glasses and lower than the average value *m_predict_* = 33.9. This trend was also corroborated in other compositions Gd_27.5_Dy_27.5_Co_10_Ni_10_Al_25_ and Pd_20_Pt_20_Cu_20_Ni_20_P_20_, underscoring the impact of high‐entropy mixing on liquid fragility.

**Figure 7:**
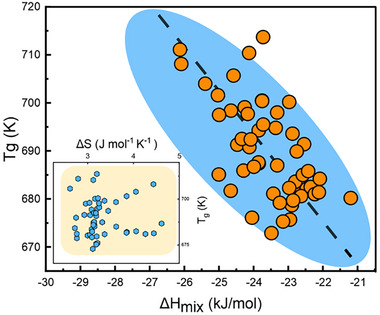
Relation between glass transition temperature *T_g_* and *ΔH_mix_*, the inset shows *T_g_* against *ΔS*.

**Figure** **7** shows the glass transition temperature *T_g_* against *ΔH_mix_* and *ΔS* for the (Cu_50_Zr_50_)_100‐_ *_x_* X*_x_* system. The data reveal a correction between *T_g_* and *ΔH_mix_*, while no apparent correlation can be found with *ΔS*. This is reasonable, considering that *T_g_* is primarily influenced by the interactions between elements rather than the entropy changes.

## 4 Discussion

The relation between fragility and entropy might be understood from an empirical Wang‐Angell formula,^[^ ^34^, ^47^ ^]^ which states that *m*  =  56Δ*C_p_* × *T_g_*/*H_m_* where *H_m_* is the melting enthalpy. If we use the empirical relation that *T_g_* = 2*T_m_*/3 for glass formers and consider *H_m_*/*T_m_* = *ΔS_m_*, then we have *m*∝Δ*C_p_* /Δ*S_m_*. Considering that the mixing entropy can be added to the melting entropy, we have δm∝  − δΔS/ (ΔS_m_)^2^. Furthermore, considering that the change in melting entropy is small in the micro‐alloying experiments, it can be approximated as constant for initial analysis. This leads to a simplified model with a negative linear relationship δ*m*∝  − δΔ*S* between the entropy‐suppressed fragility and the increase in entropy due to micro‐alloying. As shown in Figure 5, our data align with this theoretical consideration within experiment uncertainties. One can also see from Adam‐Gibbs formula: $\eta =\eta_{0}\;exp\left(\frac{A}{TS_{c}}\right)$,^[^ ^48^ ^]^ which predicts a dependence of viscosity (η)on the congurational entropy (S_c_). The viscosity of the liquid increases sharply as S_c_ decreases. This suggests that, from a thermodynamic perspective, the glass transition is the process of the freezing of the liquid entropy when the temperature is near T*_g_*. Therefore, metallic glasses with higher S_c_should exhibit less dramatic viscosity changes, which means a reduction in fragility.

We can also comprehend this result in the potential energy landscape (PEL), for our binary system, there are lots of degenerate energy levels. With the occurrence of microdoping, these degenerate energy levels will undergo significant differentiation due to the increase in mixing entropy. If the distortion caused by atomic size mismatch is considered, these differentiated energy levels will further diverge. These differentiated energy levels raise the number of accessible microstates during the cooling, enhancing the diversity of atomic arrangements. This allows atoms to adjust within a larger configurational space, thereby reducing the constraints of structural changes on dynamics, leading to a reduction in fragility.

It is important to recognize that while fragility is a measure of the dynamics of the glass transition, entropy is fundamentally a thermodynamic property. The observed correlation between these two aspects underscores the importance of entropy in not only the equilibrium state but also the kinetics of material dynamics. The results also lend credence to the thermodynamic basis of the glass transition, aligning with theories such as the random first‐order transition theory.^[^ ^49^, ^50^ ^]^ This theory posits that the configurational entropy underlies the kinetic arrest observed in the glass transition, thereby connecting the thermodynamic perspective with the dynamic behavior of glass‐forming systems.

Our results could have broad implications for the materials design. We note that the fragility concept, while initially introduced for liquid properties, has been suggested to correlate with a wide spectrum of solid glass properties, including glass‐forming ability, mechanical deformability,^[^ ^51^ ^]^ elastic properties, heat capacity changes at the glass transition, the Boson peak related to vibrational characteristics, low‐temperature heat capacity, surface diffusion, and the formation of ultra‐stable glasses. It is also crucial in glass processing such as casting, annealing, and aging. The high entropy micro‐alloying approach can adjust fragility with minimal enthalpic effects. Besides, considering the relationship between glass‐forming liquids and glass‐forming ability (GFA), it should also contribute to the development of glasses with enhanced GFA.^[^ ^52^, ^53^ ^]^

Finally, we remark that although this work focuses on metallic glass forming liquids, the general thermodynamics arguments could also be relevant to other materials. In the case of microalloying, the entropy is dominant over enthalpy. Different materials classes might have their unique composition range of *x* defining the microalloying.

## 5 Conclusion

In summary, we have shown that mixing entropy impacts the liquid dynamics through the fragility index. The results have been obtained through high entropy micro‐alloying experiments on metallic glass‐forming liquids. Our findings elucidate the distinct role of mixing entropy in liquid dynamics, particularly its influence on the fragility index of metallic glass‐forming liquids. The high‐entropy micro‐alloying approach could serve as a tool in materials science, enabling the fine‐tuning of material properties through entropy manipulation.

## Conflict of Interest

The authors declare no conflict of interest.

## Supporting information



Supporting Information

## Data Availability

The data that support the findings of this study are available in the supplementary material of this article.
