# Supplementary material for: Separating the Role of Mixing‐Entropy on the Dynamics of Glass‐Forming Liquids
Source: Adv Sci (Weinh). 2025 Apr 7;12(26):2502568. doi: 10.1002/advs.202502568 (PMC12244498; doi:10.1002/advs.202502568)
Supplement: Supplementary file 1 — Supporting Information [file ADVS-12-2502568-s001.docx]

**Supplemental Information for:**

**Separating the Role of Mixing-Entropy on the Dynamics of Glass-forming Liquids**

**1.Fragility Determining Details**

To determine fragility, the as-cast sample was heated at a selected heating rate to reach a temperature T*, which is in the supercooled liquid region. We ensure T* is above T_g_ but below T_x_. The sample was then cooled back to the glassy state using a cooling rate of Q_c_. This heatingcooling cycle was used to eliminate the thermal history and yield a glass with a consistent fictive temperature.As shown in Fig. S1, compared to the as-cast sample (the gray curve), the red and blue heat flow curves both eliminate the thermal history. However, the blue heat flow curve exhibits premature crystallization due to an excessively high chosen T*, leading to an underestimated T_g_ value. Therefore, we ultimately adopt the red curve to obtain T_g_ . For each sample, We will first attempt heating to different temperatures for several times to determine the most suitable T*. Then, this procedure will be repeated at different heating rates. We aim for the widest possible range of heating rates within the limitations of equipment reliability. And for our DSC, the range of reliable heating rates is typically between 5 K/min and 100 K/min. For Flash DSC, the range of reliable heating rates is typically between 50 K/s and 500 K/s.

By repeating the above steps, we record T_g_ as a function of heating rate (Q). We fitted the fragility with an equation as suggested by Wang et al

$${log}_{10}(Q/Q_{s})=m-m(T_{f}^{s}/T_{f})$$

Here $T_{f}$ = $T_{g}$, $T_{f}^{s}$ represents the $T_{g}$ at a standard heating rate Q_s_. In our work , we select Q_s_ = 20 K/min.


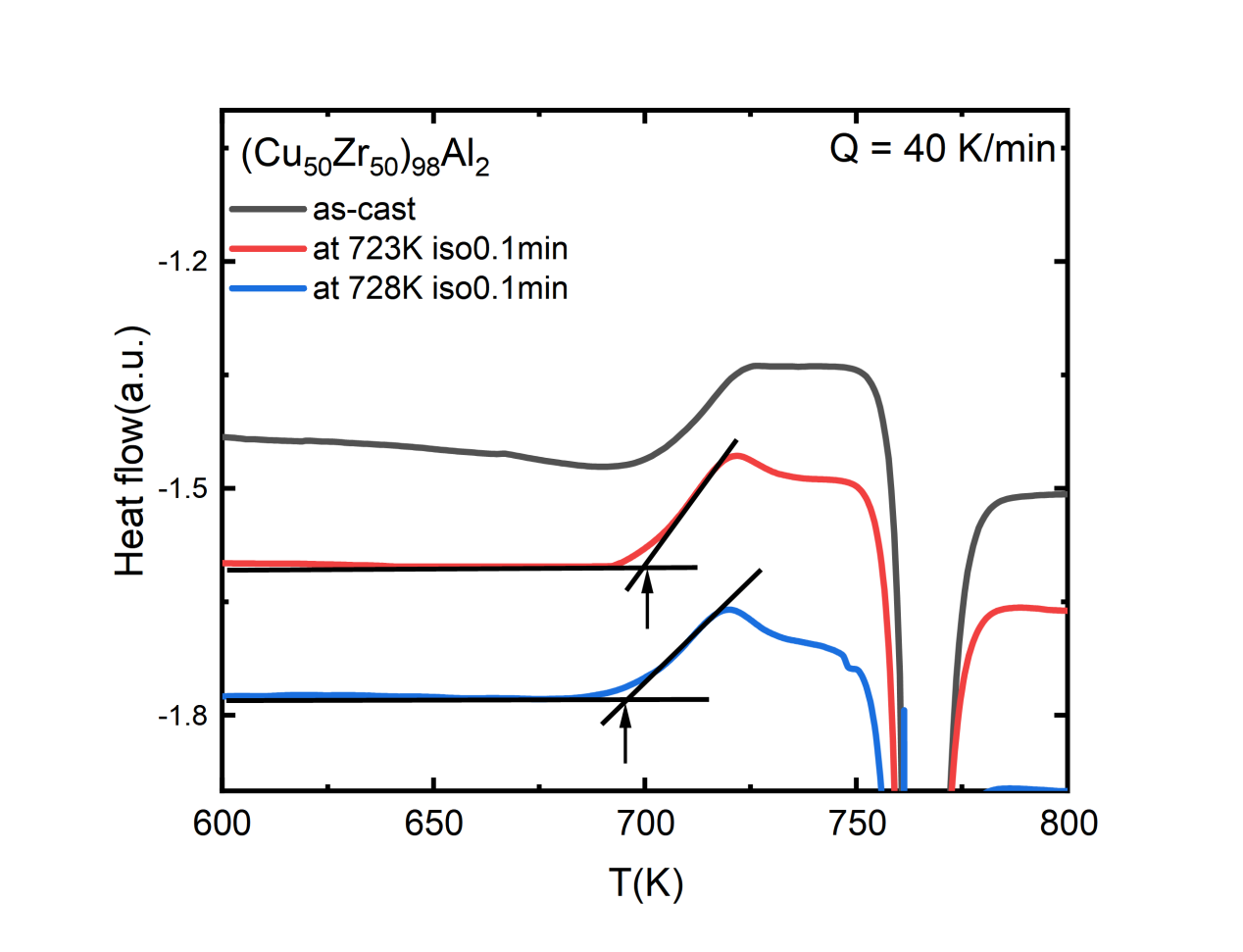


**Figure S1.**Heat flow curves for T* determining.

**2.X-ray diffraction patterns**


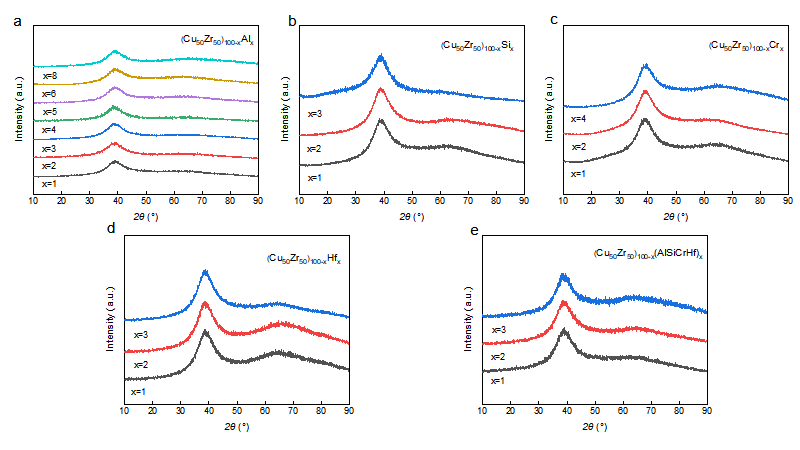


**Figure S2.** X-ray diffraction patterns for (Cu_50_Zr_50_)_100-x_X_x_ **(a)** X = Al, **(b)** X = Si, **(c)** X = Cr **(d)** X = Hf **(e)** X = AlSiCrHf


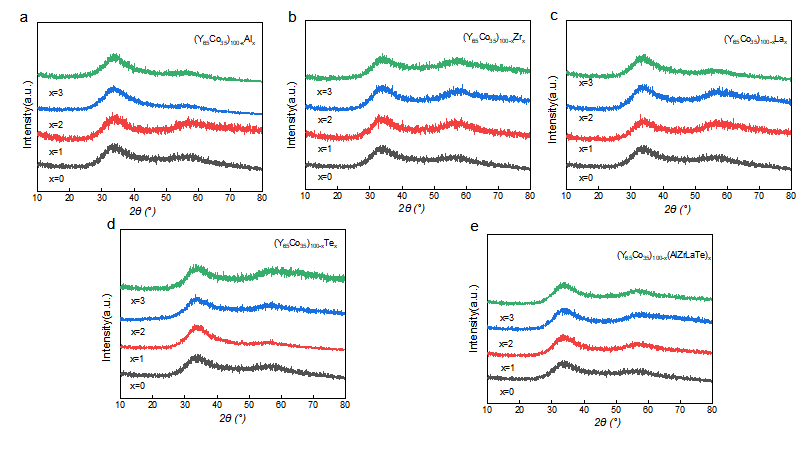


**Figure S3.** X-ray diffraction patterns for (YCo_65_Co_35_)_100-x_X_x_ **(a)** X = Al, **(b)** X = Xr, **(c)** X = La **(d)** X = Te **(e)** X = AlZrLaTe

1. **Microstructure of the CuZr-based MGs**


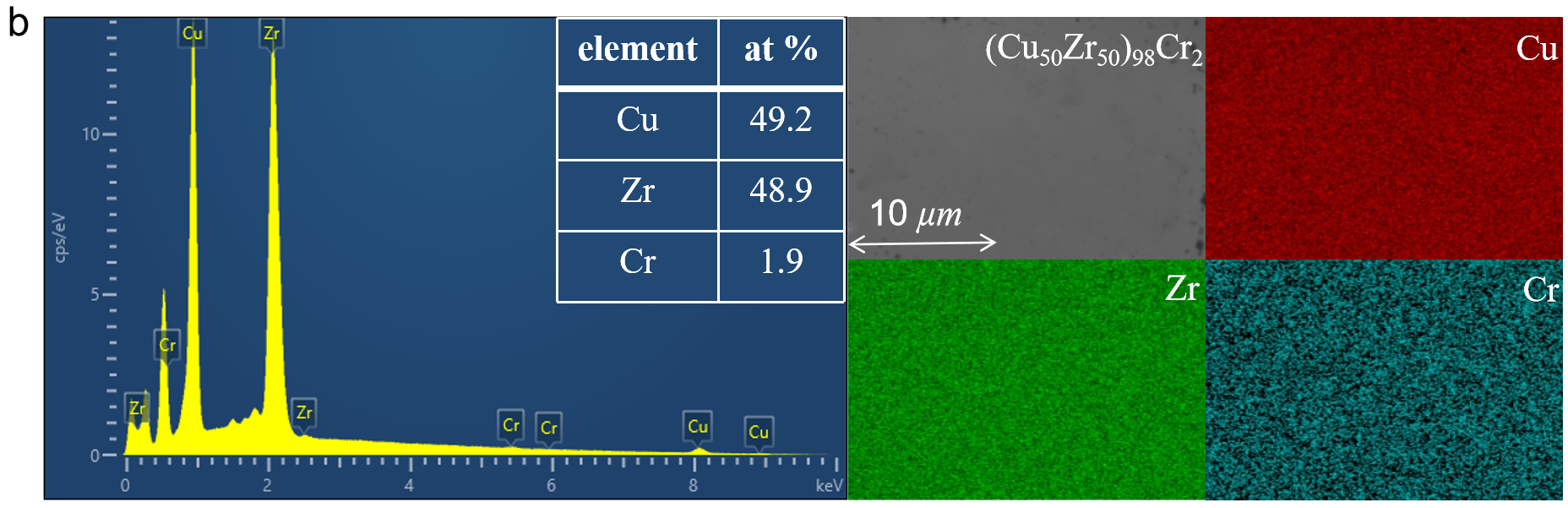

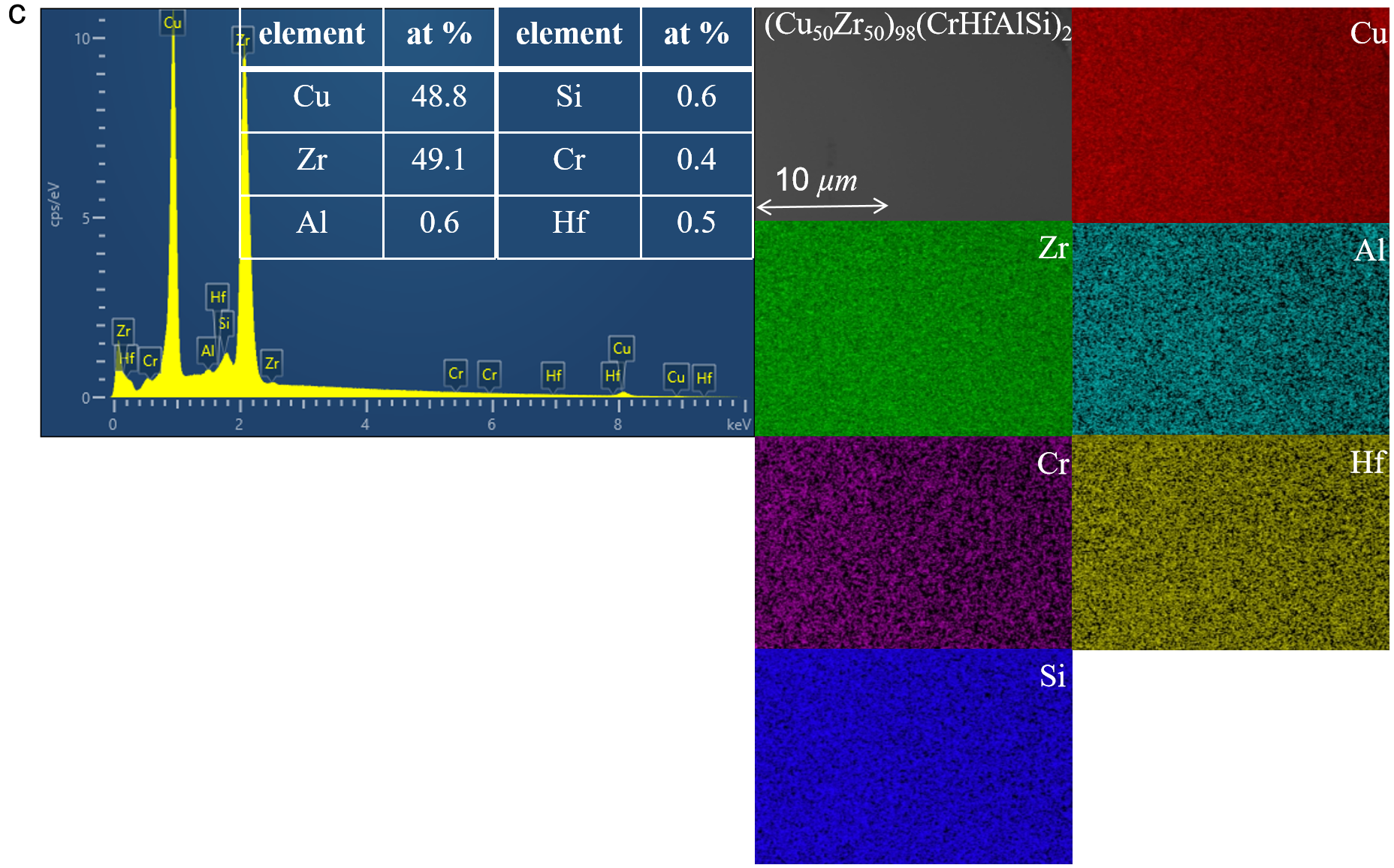

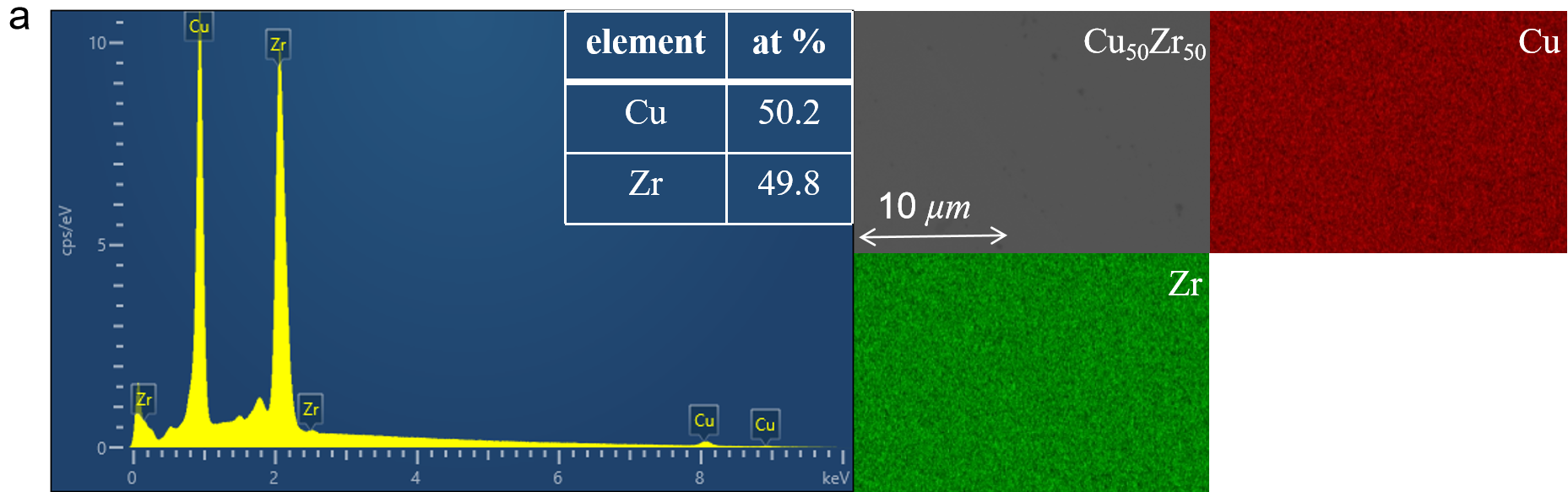


**Figure S4.** Microstructure of the CuZr-based MGs. SEM images, element content and corresponding EDS mapping of **(a)** Cu_50_Zr_50_ sample, **(b)** (Cu_50_Zr_50_)_98_Cr_2_ sample, and **(c)** (Cu_50_Zr_50_)_98_(AlSiCrHf)_2_ sample.

**4. Heat flow curves and fitted curves**


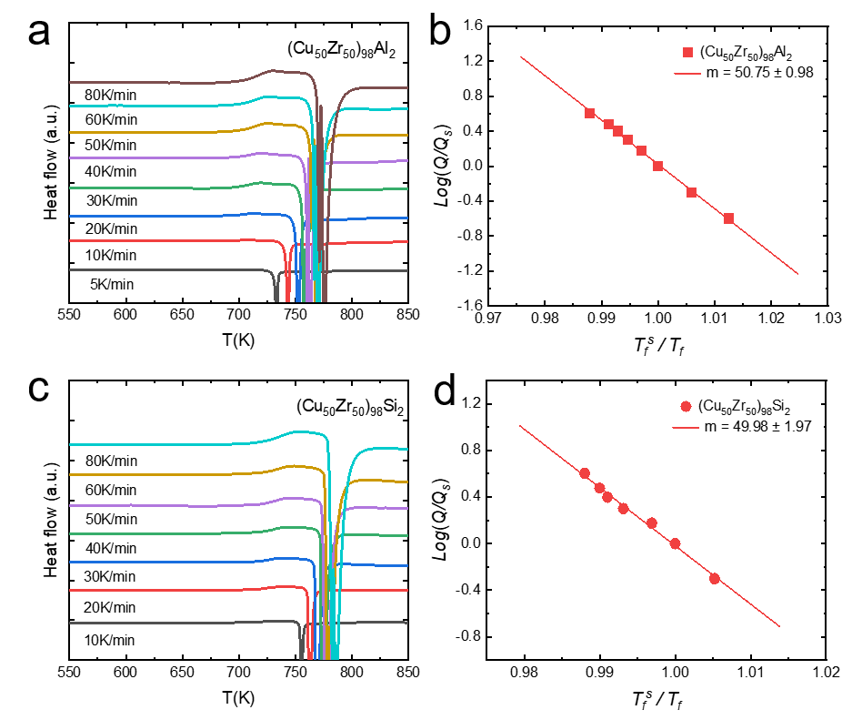

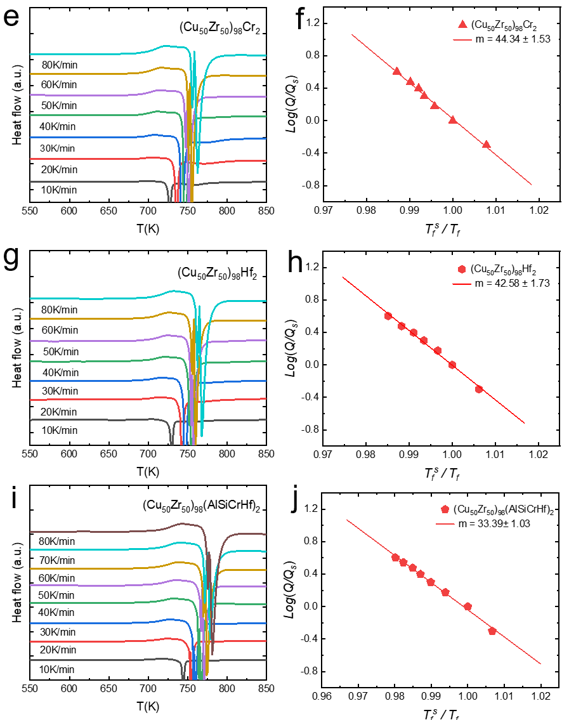


**Figure S5.** Curves for fragility determining of (Cu_50_Zr_50_)_98_X_2_. **(a,c,e,g,i)** are DSC heat flows curves. **(b,d,f,h,j)** are fitted curves.


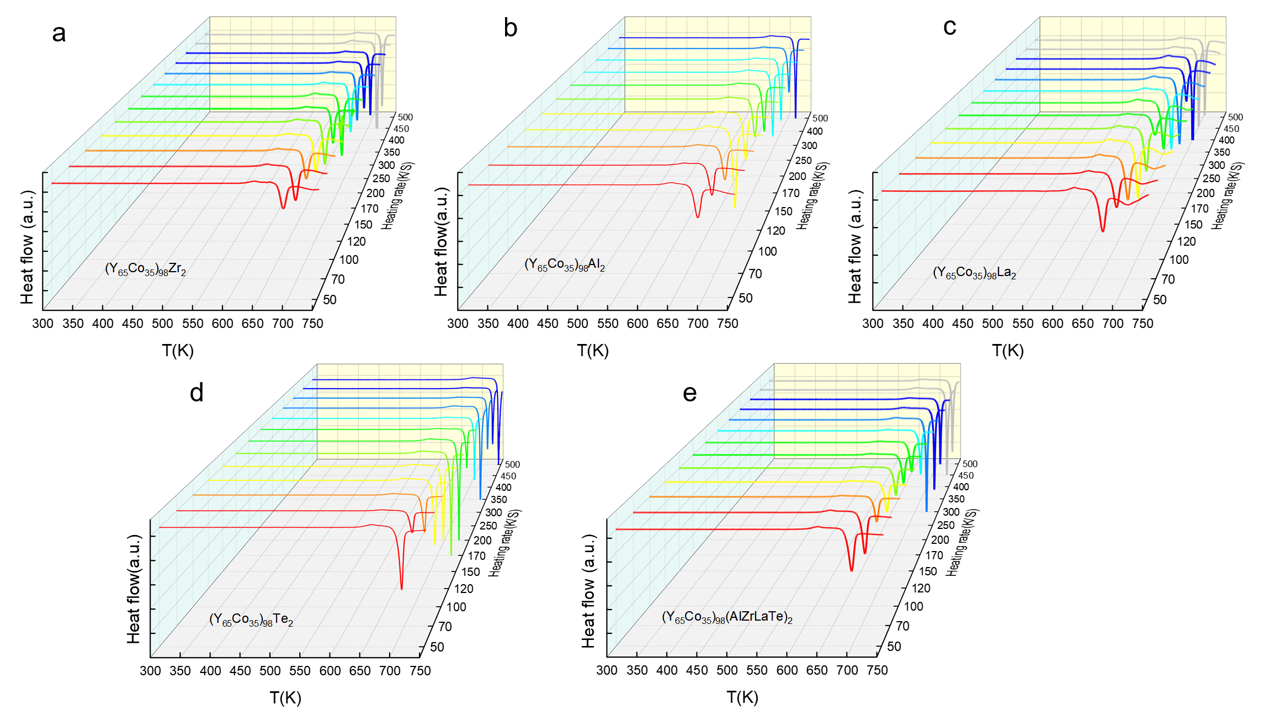


**Figure S6.** FSC heat flow curves of (Y_65_Co_35_)_98_X_2_ MG samples. (a) X = Zr (b) X = Al (c) X =La (d) X = Te (e) X = AlZrLaTe


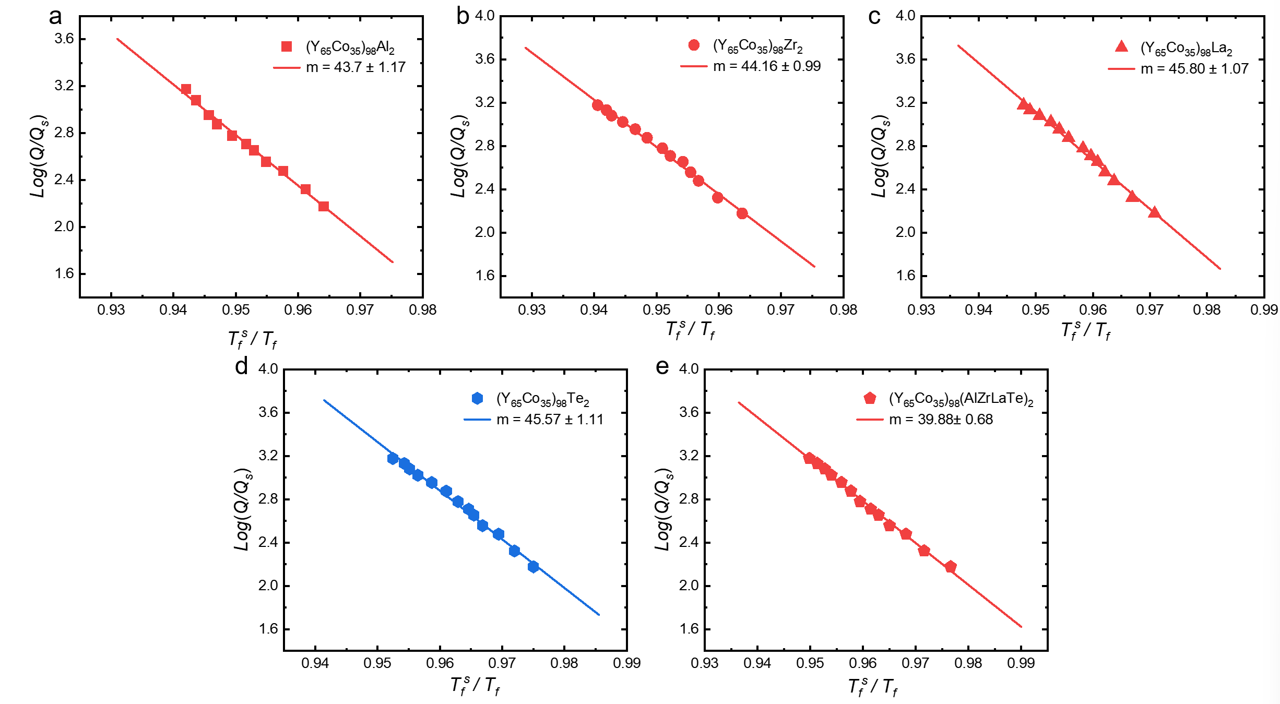


**Figure S7.** Fitted curves of (Y_65_Co_35_)_98_X_2_ MG samples. (a) X = Zr (b) X = Al (c) X =La (d) X = Te (e) X = AlZrLaTe

1. **Fitted Data and Curves for (Cu_50_Zr_50_)_98_(AB/ABC/ABCD)_2_ MGs**


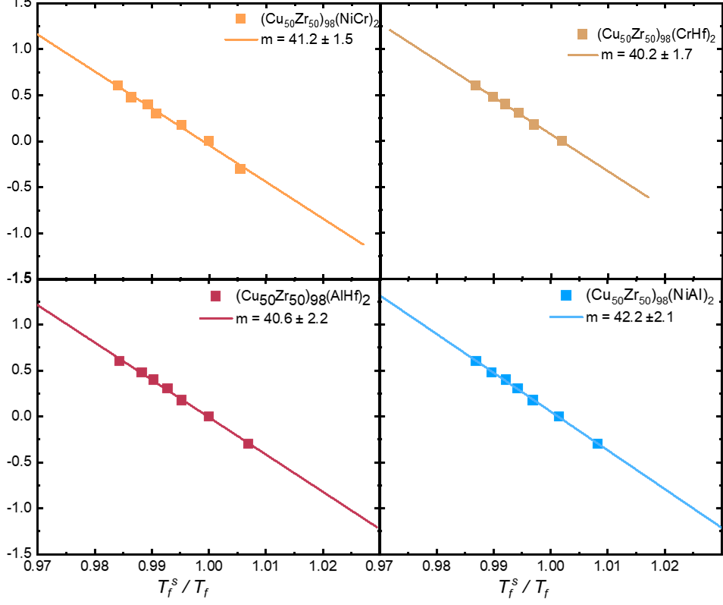

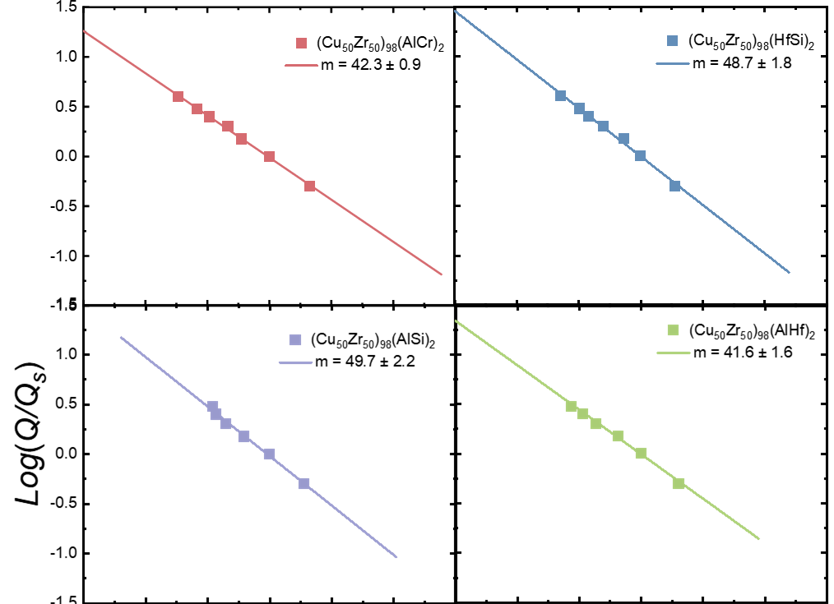


**Figure S8.** Fitted Curves of the (Cu_50_Zr_50_)_98_(AB)_2_ MG samples. A, B = Al, Si, Cr, Hf，Ni and each elements having the equal molar fraction.


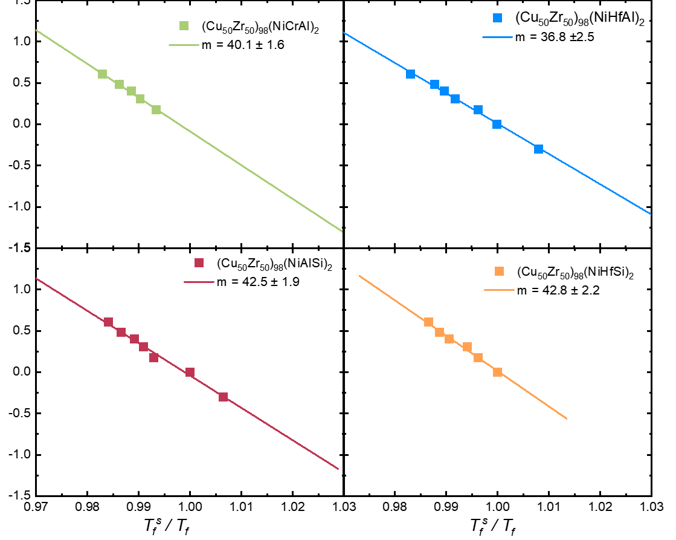

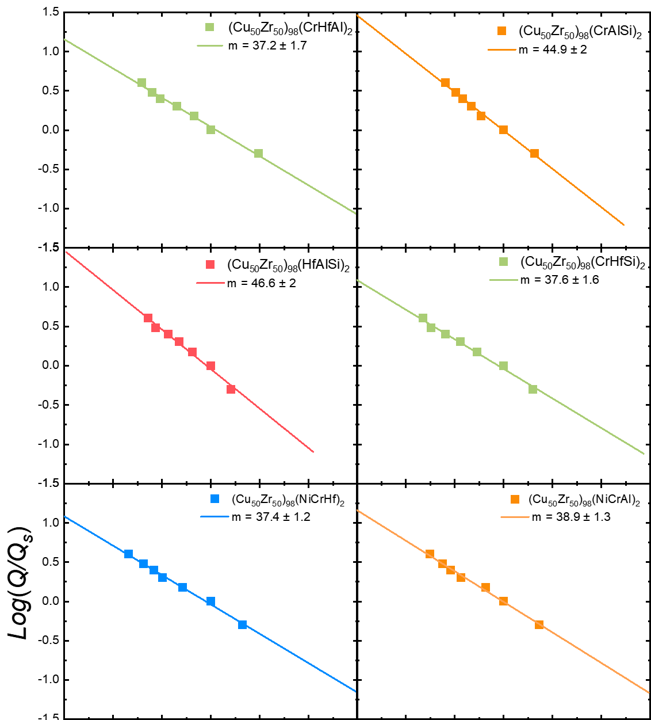


**Figure S9.** Fitted Curves of the (Cu_50_Zr_50_)_98_(ABC)_2_ MG samples. A, B, C = Al, Si, Cr, Hf，Ni and each elements having the equal molar fraction.


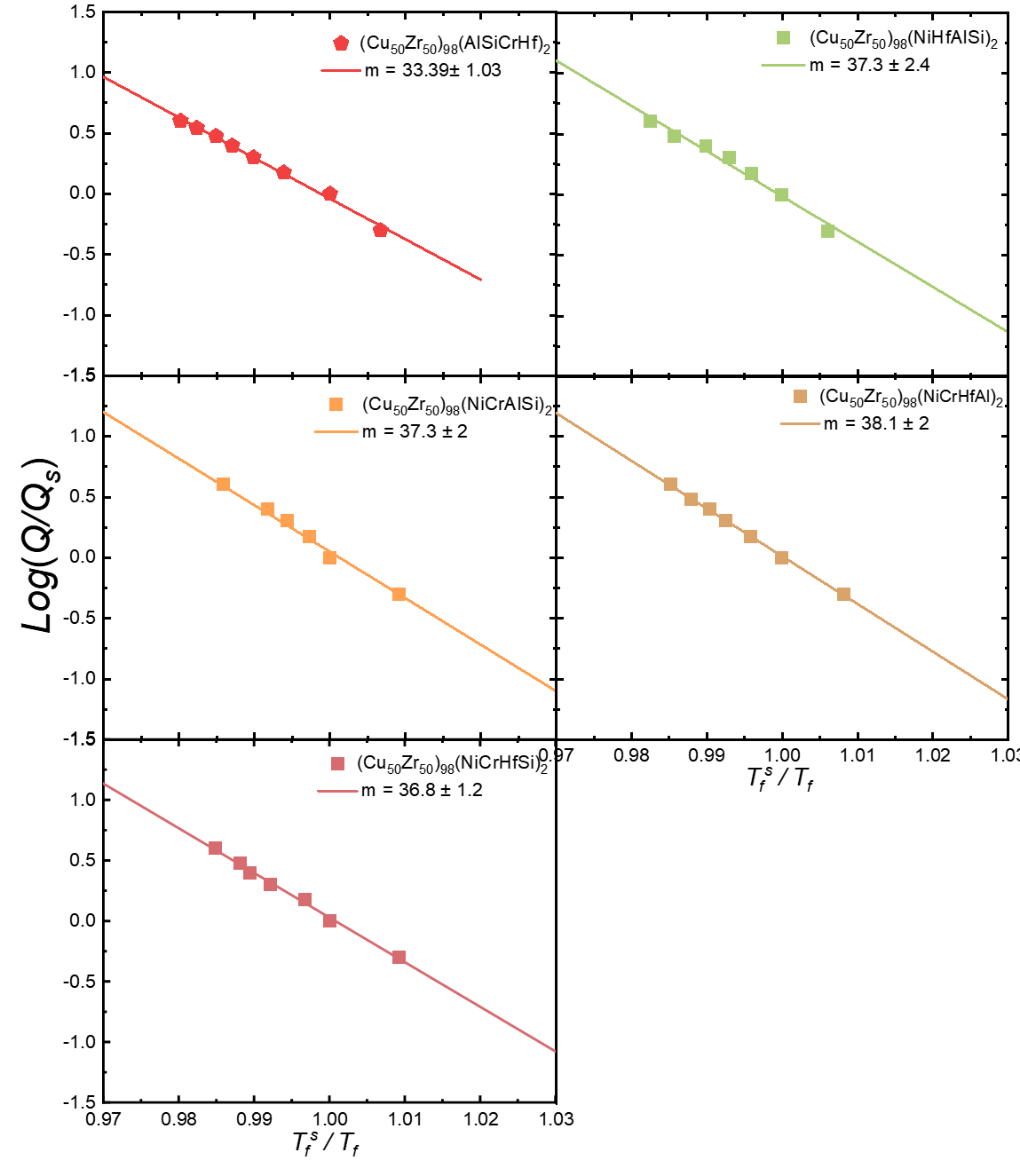


**Figure S10.** Fitted Curves of the (Cu_50_Zr_50_)_98_(ABCD)_2_ MG samples. A, B, C，D = Al, Si, Cr, Hf，Ni and each elements having the equal molar fraction.

1. **Fitted Data and Curves for HEMGs**


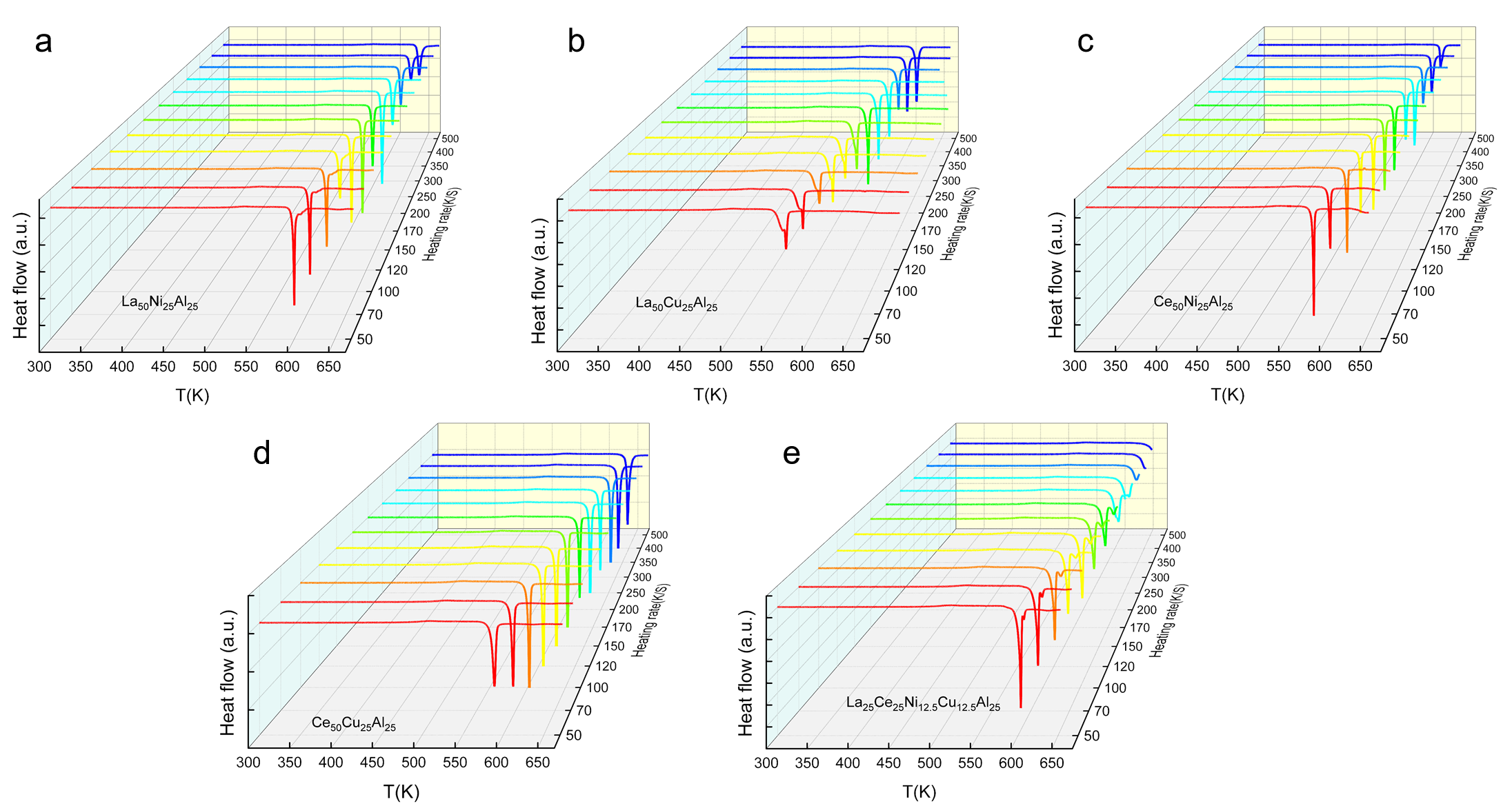


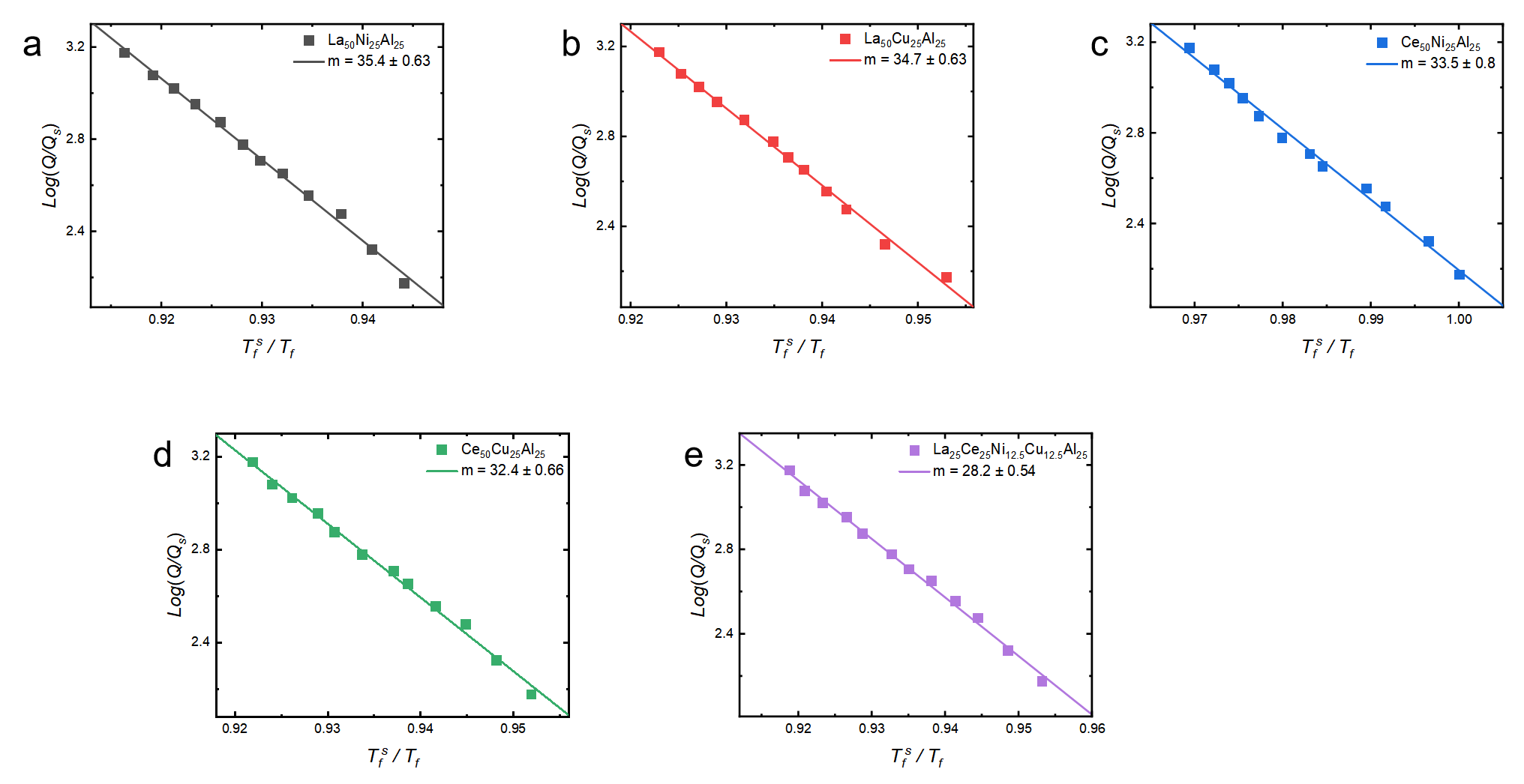
**Figure S11.** FSC heat flow curves of La/Ce-Cu/Ni-Al samples. **(a)** La_50_Ni_25_Al_25_ **(b)** La_50_Cu_25_Al_25_ **(c)** Ce_50_Ni_25_Al_25_ **(d)** Ce_50_Cu_25_Al_25_ **(e)** La_25_Ce_25_Cu_12.5_Ni_12.5_Al_25_

**Figure S12.** Fitted curves of La/Ce-Cu/Ni-Al samples. **(a)** La_50_Ni_25_Al_25_ **(b)** La_50_Cu_25_Al_25_ **(c)** Ce_50_Ni_25_Al_25_ **(d)** Ce_50_Cu_25_Al_25_ **(e)** La_25_Ce_25_Cu_12.5_Ni_12.5_Al_25_


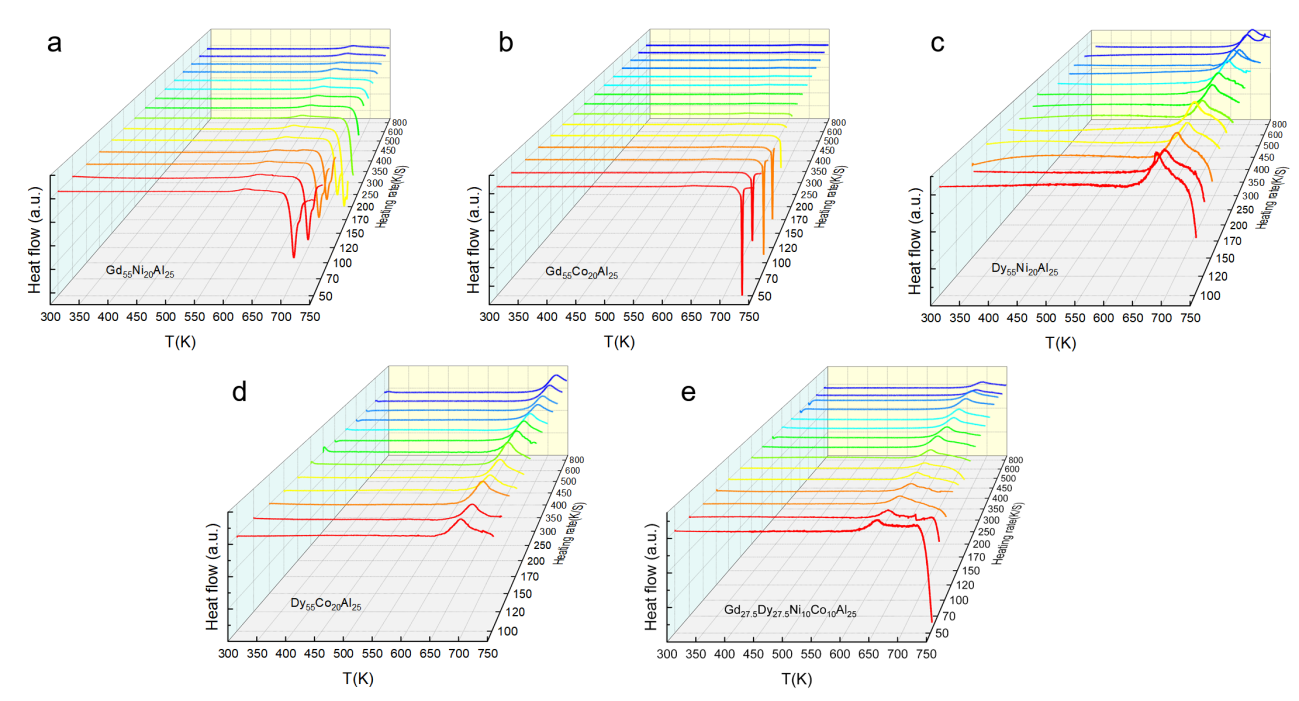


**Figure S13.** FSC heat flow curves of Gd/Dy-Ni/Co-Al samples. **(a)** Gd_55_Ni_20_Al_25_ **(b)** Gd_55_Co_20_Al_25_ **(c)** Dy_55_Ni_20_Al_25_ **(d)** Dy_55_Co_20_Al_25_ **(e)** Gd_27.5_Dy_27.5_Ni_10_Co_10_Al_25_


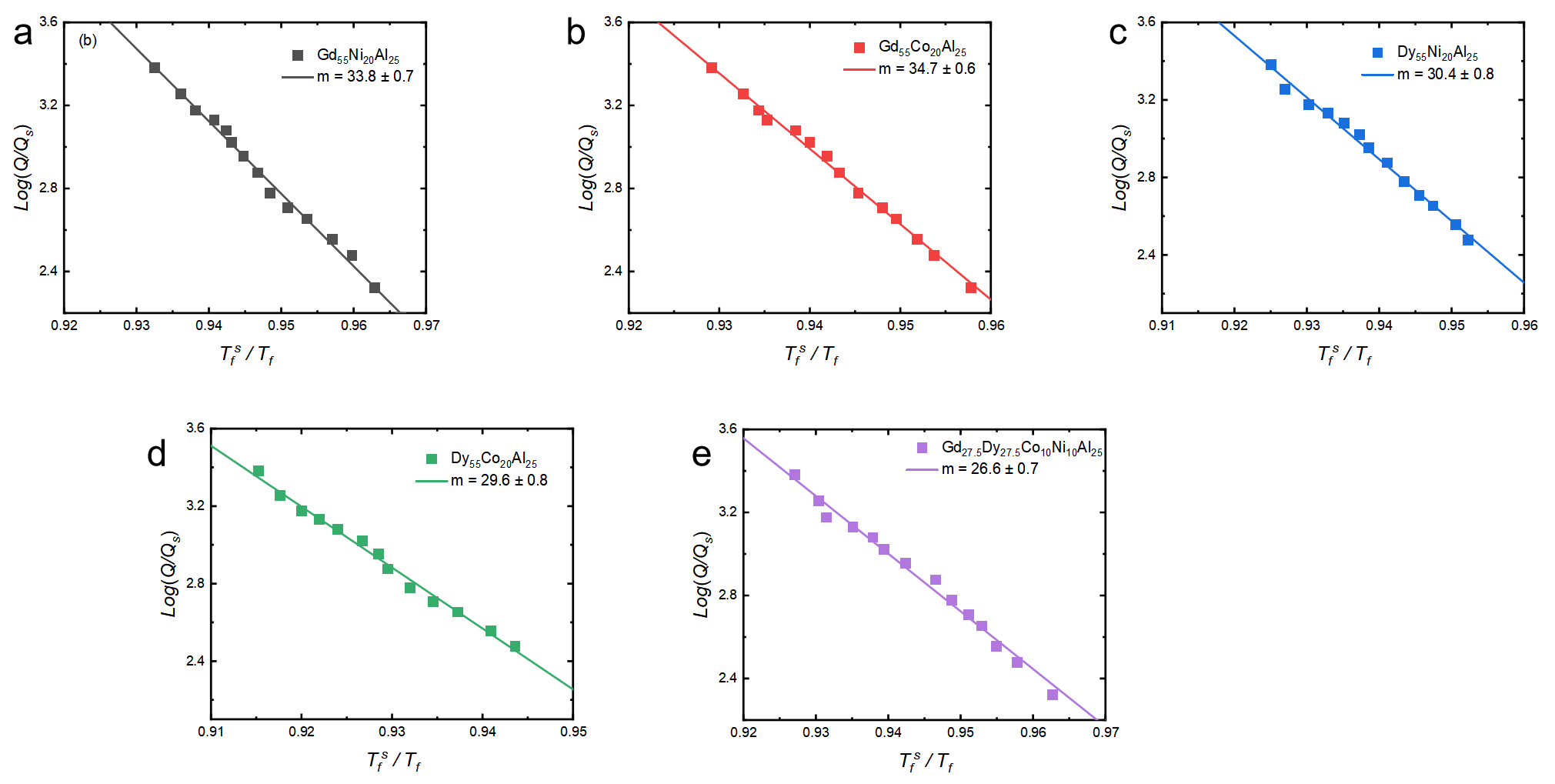


**Figure S14.** Fitted curves of Gd/Dy-Ni/Co-Al samples. **(a)** Gd_55_Ni_20_Al_25_ **(b)** Gd_55_Co_20_Al_25_ **(c)** Dy_55_Co_20_Al_25_ **(d)** Dy_55_Ni_20_Al_255_ **(e)** Gd_27.5_Dy_27.5_Ni_10_Co_10_Al_25_


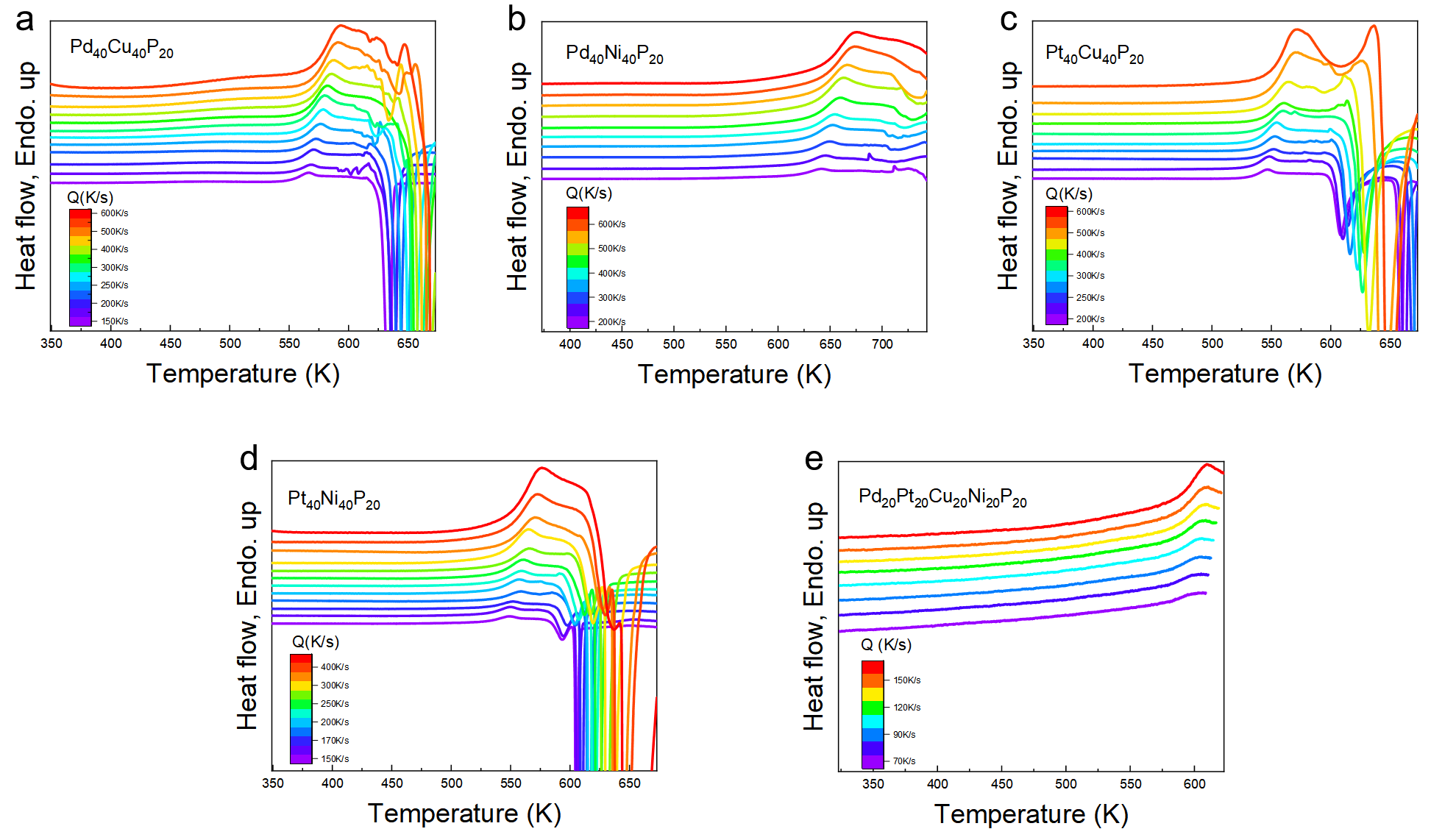


**Figure S15.** FSC heat flow curves of Pd/Pt-Cu/Ni-P samples. **(a)** Pd_40_Cu_40_P_20_ **(b)** Pd_40_Ni_40_P_20_ **(c)** Pt_40_Cu_40_P_20_ **(d)** Pt_40_Ni_40_P_20_ **(e)** Pd_20_Pt_20_Cu_20_Ni_20_P_20_


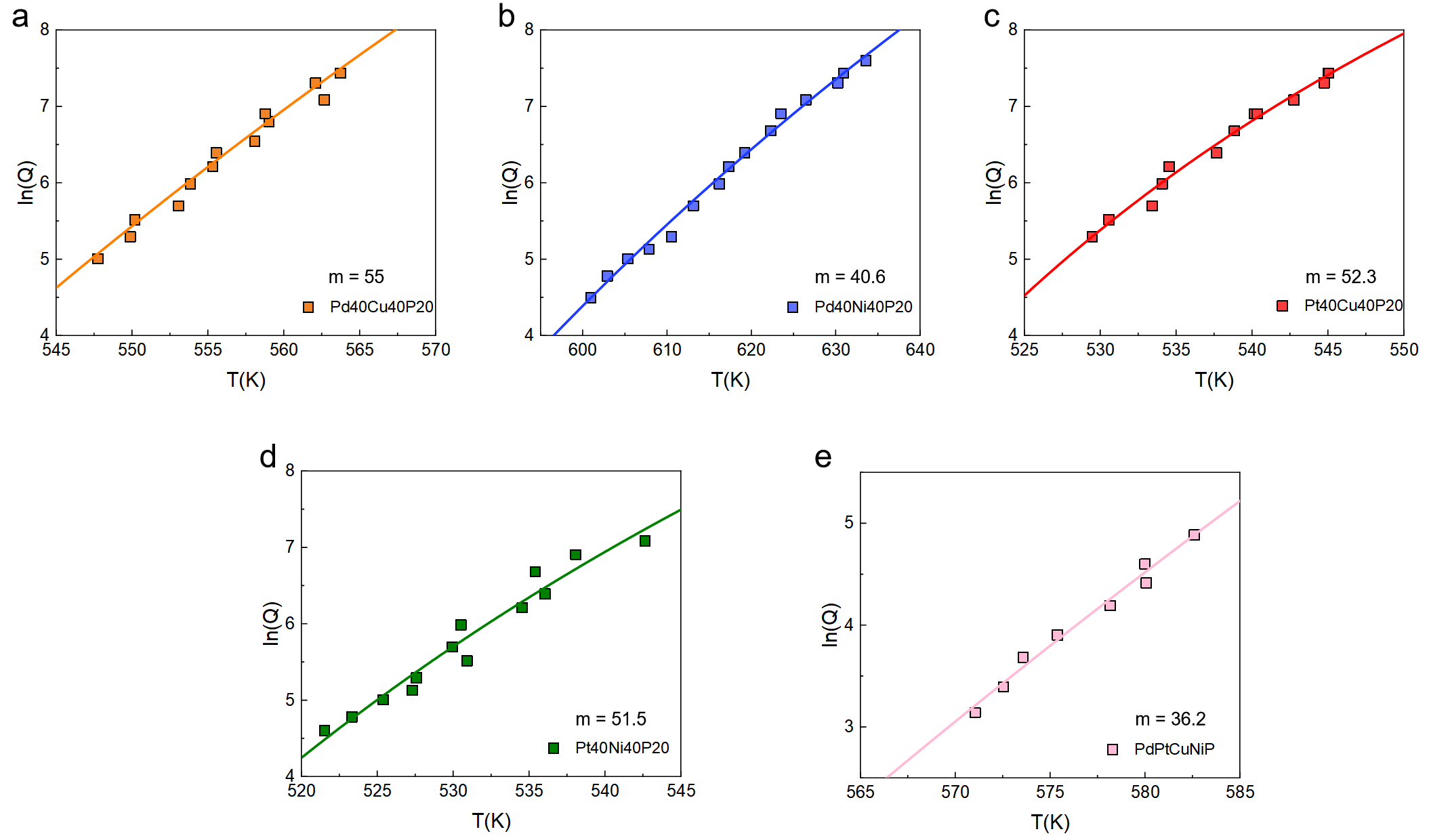


**Figure S16.** VFT fitted curves of Pd/Pt-Cu/Ni-P samples. **(a)** Pd_40_Cu_40_P_20_ **(b)** Pd_40_Ni_40_P_20_ **(c)** Pt_40_Cu_40_P_20_ **(d)** Pt_40_Ni_40_P_20_ **(e)** Pd_20_Pt_20_Cu_20_Ni_20_P_20_

(To maintain consistency with earlier studies, the fragility of the Pd/Pt-based metallic glasses was fitted using the Vogel-Fulcher-Tammann (VFT) method.)

1. **Atomic size mismatch effect**


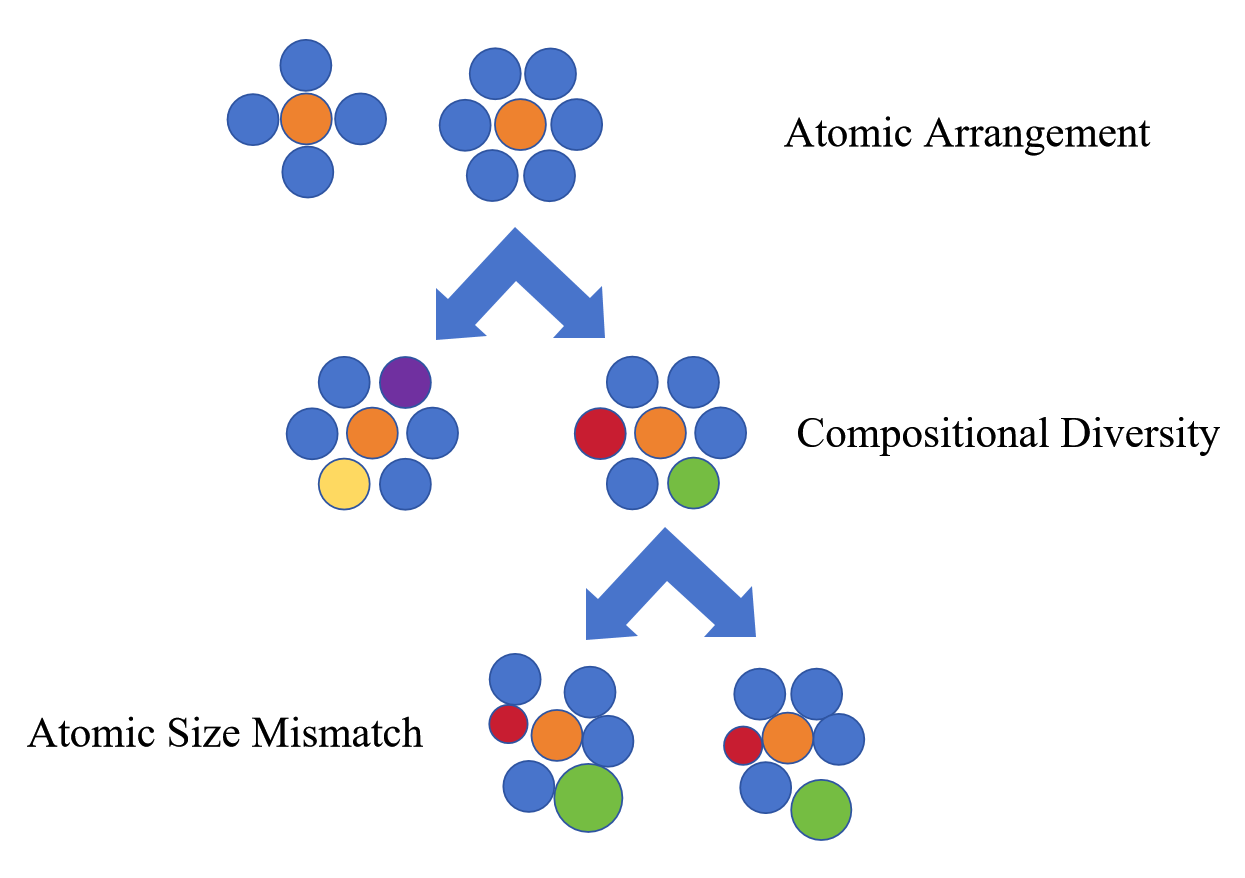


**Figure S17.** A schematic description for degenerate energy levels undergo significant differentiation caused by compositional diversity and atomic size mismatch.

One of the four core effects of high-entropy alloys is lattice distortion. However, one can easily imagine that simply increasing configurational entropy does not significantly enhance the distortion effect, but rather provides more pronounced chemical disorder . Instead, Only a significant change in atomic size can enhance the distortion effect. In this case, it is easy to envision that, based on the increase in mixing entropy, atomic size mismatch will further differentiate the degenerate configurations. Thus, in highly concentrated alloys, chemical effect (related to compositional diversity)and atomic size effect are often considered to work together, exerting a combined influence.

However, it should be noted that in our binary micro-alloying system, the effect of atomic size mismatch may be less pronounced due to the overwhelming presence of the primary atoms. As evidence, we can compare the change in entropy from Cu50Zr50 to (Cu50Zr50)98(CrHfAlSiNi)2. The configurational entropy increased from 5.76 to 6.73 J/mol K (a 17% increase), whereas, the , related to the atomic size mismatch and the atomic packing changed from -3.44 to -3.48 J/mol K, with only about 1% change.
